# On the Impact of Chemo-Mechanically Induced Phenotypic Transitions in Gliomas

**DOI:** 10.3390/cancers11050716

**Published:** 2019-05-24

**Authors:** Pietro Mascheroni, Juan Carlos López Alfonso, Maria Kalli, Triantafyllos Stylianopoulos, Michael Meyer-Hermann, Haralampos Hatzikirou

**Affiliations:** 1 Braunschweig Integrated Centre of Systems Biology and Helmholtz Center for Infectious Research, 38106 Braunschweig, Germany; pietro.mascheroni@helmholtz-hzi.de (P.M.); juancarlos.lopezalfonso@helmholtz-hzi.de (J.C.L.A.); 2 Department of Gastroenterology, Hepatology and Endocrinology, Hannover Medical School, 30625 Hannover, Germany; 3 Cancer Biophysics Laboratory, Department of Mechanical and Manufacturing Engineering, University of Cyprus, 1678 Nicosia, Cyprus; kalli.maria@ucy.ac.cy (M.K.); tstylian@ucy.ac.cy (T.S.); 4 Centre for Individualized Infection Medicine, 30625 Hannover, Germany; 5 Institute for Biochemistry, Biotechnology and Bioinformatics, Technische Universität Braunschweig, 38106 Braunschweig, Germany

**Keywords:** glioma, solid stress, phenotypic transitions, stress-alleviation therapy

## Abstract

Tumor microenvironment is a critical player in glioma progression, and novel therapies for its targeting have been recently proposed. In particular, stress-alleviation strategies act on the tumor by reducing its stiffness, decreasing solid stresses and improving blood perfusion. However, these microenvironmental changes trigger chemo–mechanically induced cellular phenotypic transitions whose impact on therapy outcomes is not completely understood. In this work we analyze the effects of mechanical compression on migration and proliferation of glioma cells. We derive a mathematical model of glioma progression focusing on cellular phenotypic plasticity. Our results reveal a trade-off between tumor infiltration and cellular content as a consequence of stress-alleviation approaches. We discuss how these novel findings increase the current understanding of glioma/microenvironment interactions and can contribute to new strategies for improved therapeutic outcomes.

## 1. Introduction

Gliomas originate from glial cells or their precursors in the brain and constitute the most common type of malignant brain tumor in adults [1,2]. These tumors display large heterogeneities at multiple levels, i.e., clinical, histological and genetical. Until recently, glioma classification was mostly based on microscopic examination of histological sections by pathologists, distinguishing tumours according to their microscopic similarities to different glial cell types [3,4]. Currently, the 2016 World Health Organization (WHO) classification of tumours of the central nervous system integrates molecular biomarkers and histological features to categorize gliomas into different grades [3]. The infiltrative nature of gliomas is considered one of the main reasons for poor treatment outcomes. Despite recent progresses in neuro-oncology and advances in medical imaging, complete tumor eradication is often unsuccessful, leading to tumor recurrence in most of the cases [5]. A better understanding of the mechanisms underlying glioma progression is urgently needed to develop effective treatments, and reduce glioma associated mortality.

In the last few years, it has become increasingly clear that tumor microenvironment plays a critical role in tumor progression and resistance to therapies [6,7,8,9,10]. Two important components characterize the tumor microenvironment, namely chemical and mechanical factors. A major role amongst the first is played by oxygen, and particularly by its deficiency (hypoxia), which correlates with tumor invasiveness and malignancy [11]. Regarding the physical environment, the growth of a tumor in the confined space of a tissue is known to generate mechanical forces [12,13]. These forces may hinder cell proliferation, induce apoptosis, enhance cellular invasive potential and lead to blood vessel occlusion [12,14,15,16]. In response to chemical and physical stimuli, tumor cells exhibit phenotypic changes. A prominent phenotypic plasticity mechanism is related to the mutually exclusive switching between the proliferating and migratory phenotypes. This phenomenon, also known as the Go-or-Grow (GoG) mechanism, has been suggested to impact the invasive potential of gliomas [17,18,19,20,21,22]. The interplay between tumor behavior and microenvironmental stimuli is being actively investigated, and it is becoming the target of novel therapeutic strategies [23]. For instance, vascular normalization and stress-alleviation approaches have been investigated to treat solid tumors [24]. Stress-alleviation therapies propose to reduce mechanical stresses within the tumor to decompress tumor blood vessels and thus, improve tissue perfusion and the delivery of chemotherapeutic agents. This is achieved by targeting specific components of the tumor microenvironment, such as collagen and hyaluronan [12]. Depletion of these constituents by repurposing of approved drugs with anti-fibrotic properties has indeed showed to reduce tumor stiffness and mechanical stresses, increase tissue hydraulic conductivity and decompress tumor blood and lymphatic vessels [25,26,27]. However, due to the complexity of the tumor–microenvironment interplay, success rates of those normalization therapies are largely variable and still under intense investigation.

Mathematical modeling is emerging as a complimentary methodology to aid biologists and clinicians in the understanding of tumor-surrounding tissue interactions [28,29]. Mathematical models are powerful tools to decipher the mechanisms underlying glioma infiltration and progression, as well as to suggest potential strategies for therapeutic interventions [4]. In addition, they help bridging the knowledge gap between microenvironmental changes and cellular plasticity, by testing hypotheses that would be difficult to experimentally prove. Several modeling approaches have been developed so far, with models based on discrete, continuous or hybrid descriptions of tumor-microenvironmental interactions [4,30,31,32,33,34,35,36,37,38,39].

In this work, we propose a mathematical model describing the induction of phenotypic transitions in cancer cells by both chemical and mechanical cues. The model is based on the previous work in [40], in which the authors introduced a model for glioma invasion accounting for nutrient-driven phenotypic switching. They considered tumor response to vaso-modulatory interventions, a cancer treatment that targets tumor vasculature to improve tissue perfusion. The authors found that glioma responses to such treatments fall in two regimes, depending on a critical cellular proliferation/motility ratio. In one case, the treatment reduces tumor front speed and increases tumor infiltration. In the other case, the opposite situation occurs. The aim of this study is two-fold: (i) to complement the previous model in [40] with mechanical-driven phenotypic transitions, and (ii) to evaluate the effects of a stress-alleviation treatment on glioma progression and invasion. Models describing the application of stress-alleviation treatments already exist in the literature [26]; however, to the authors knowledge, the influence of cellular phenotypic transitions—resulting from these microenvironmental treatments—on tumor progression has not been investigated yet.

## 2. Results

### 2.1. Cellular Responses to Mechanical Compression

**Figure 1:**
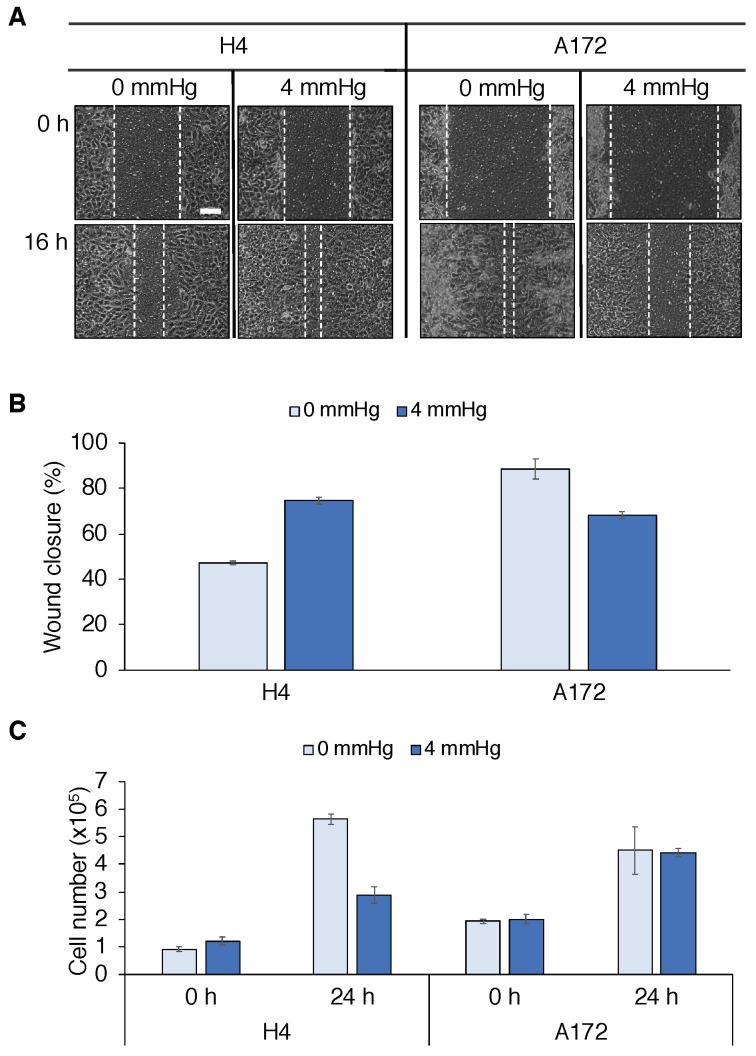
Effects of mechanical compression on glioma cell lines. (**A**) Wound closure assay performed on two different glioma cell lines (H4 and A172 cells). Cancer cells are seeded on the inner chamber of a transwell insert, on the top of which an agarose cushion is positioned. A piston with adjustable weight applies a solid stress (4 mmHg) on the cells and the effects of compression are visualized after 16 h (scale bar: 0.1 mm). (**B**) Quantification of wound closure at 16 h. For H4 cells, compression increases their migratory behavior, leading to a higher wound closure percentage. On the other hand, A172 cells display higher migratory potential in the control case (0 mmHg), but respond to compression by reducing migration on the substrate. (**C**) An Alamar Blue assay is performed on H4 and A172 cells before and after (24 h) mechanical compression (4 mmHg) as an indicator for total cell number in the transwell insert. While compression reduces the number of H4 cells, no influence on replication is observed for A172 cells. In (**B** and **C**) error bars show standard errors.

We performed in vitro experiments to study the effects of mechanical compression on glioma cells. Results are reported in Figure 1 and the experimental procedure is described in the Materials and Methods section. We carried out a wound closure and a cell viability assay, indicative for cell number, to quantify glioma cell migration and proliferation in two different cell lines, i.e., H4 and A172 cells (ATCC). H4 cells responded to compressive stresses by increasing migration and decreasing proliferation, in line with the GoG mechanism. On the other hand, we observed that A172 cells decreased motility and did not substantially modify their cell number. These results show that tumor responses to mechanical compression were cell line-specific. Thus, we could expect in reality to have a heterogeneity of mechanical responses within the same tumor. Our goal was to highlight the role of mechanical cues in determining the tumor cell population behavior, in terms of growth and migration. Herein, we consider phenotypic transitions driven by mechanical pressure as observed in the H4 cell line, i.e., compression favors cell migration and inhibits cell proliferation. For this type of cell phenotypic transitions, we cannot intuitively extrapolate their manifestation to the tumor population level dynamics. On the contrary, the behavior of A172 cells is rather predictable without the help of mathematical modeling. Indeed, gives rise to compact-growing tumors, in which the reduction of cell motility following compression leads to limited tumor infiltration. Modeling phenotypic transitions based on the H4 cell line, on the other hand, led to non-trivial results, as we investigate in this study.

### 2.2. Mathematical Model

**Figure 2:**
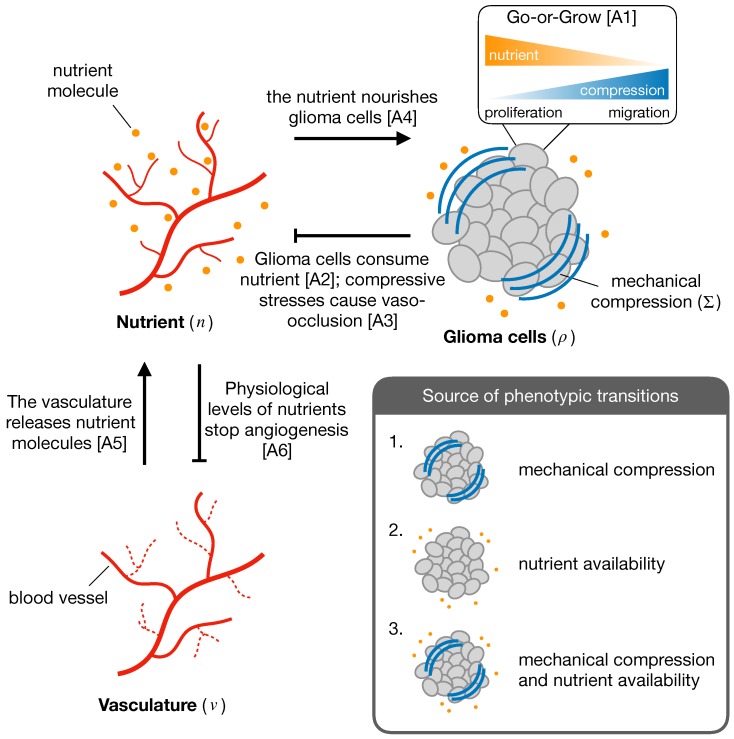
Scheme of the interactions between the different components of the system, i.e., glioma cells, nutrient availability and vasculature. The inset displays the different sources of phenotypic transitions in glioma cells considered in the model.

The mathematical model describes the growth of a vascularized tumor considering the interplay between two cell phenotypes, namely proliferative and migratory. The normalized system variables are the density of tumor cells ρ, the concentration of a nutrient, i.e., oxygen *n*, and the tumor vascular density *v*. Figure 2 shows a schematic representation of the system interactions and model assumptions, which are summarized below:

A1 Glioma cells express a proliferative or migratory phenotype depending on the microenvironmental cues. In particular, high (low) nutrient availability and low (high) mechanical compression induce a proliferative (migratory) phenotype [11,14,15,16,17,20,21].
A2 Glioma cells consume nutrient provided by the vasculature [41].
A3 Increased mechanical pressure in regions of high glioma density induce blood vessel collapse and decrease nutrient availability [42].
A4 Oxygen is essential for glioma growth and progression [11,43].
A5 Blood vessels release nutrients [43,44].
A6 The sprouting of new blood vessels stops when physiological levels of nutrients are restored [40,43].

**Figure 3:**
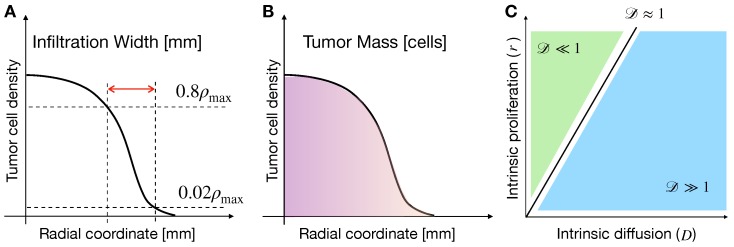
Graphical representation of model observables and glioma characteristics. Schemes representing tumor infiltration width (IW) (**A**) and tumor mass (TM) (**B**), describing tumor invasiveness and burden in the surrounding tissue, respectively. (**C**) Characteristics of gliomas across the cellular intrinsic diffusion and proliferation space. The relative importance of cell migration to proliferation is described by the non-dimensional number $D\equiv D\ell_{t}^{-2}r^{-1}$, which splits the plane in regions where proliferation (green) or migration (blue) dominate [38].

We characterize tumors by two quantities, namely the infiltration width (IW) and the tumor mass (TM), see the schematic in Figure 3A,B. Tumor IW is defined by the difference between the radial coordinates in which glioma cell density is 80% and 2% of the maximum cellular density, at the last time-step of simulations. In turn, the tumor mass is calculated by the integral

(1) $$\mathrm{TM}=4\pi \rho_{c}\int r^{2}\rho \;dr,$$

in which ρ is the tumor cell density at the last time-step of simulations, and $\rho_{c}$ is the carrying density of glioma cells. In the following sections, we investigate the dependence of IW and TM on different values of model parameters, as well as on different mechanisms driving cell proliferation and migration. Model results are presented as simulation maps showing the variations of the observable quantities over the (D,r) space, i.e., the parameter space of cellular intrinsic diffusion (*D*) and proliferation (*r*). Such space has been reported helpful for categorizing different glioma behaviors [38]. Indeed, by defining the non-dimensional number $D\equiv D\ell_{t}^{-2}r^{-1}$, in which $\ell_{t}$ is the characteristic tumor length (≈1cm), it is possible to partition the pathophysiological (D,r) space in two distinct regions, see Figure 3C. In particular, for D≪1, tumors are characterized by a dominant proliferative behavior, whereas for regions in which D≫1 tumors display a more invasive behavior.

### 2.3. Compression-Driven Phenotypic Transitions

We started our analysis by considering compression-driven phenotypic transitions. In this way, the evolution of the tumor was decoupled from the nutrient and vasculature equations—see the Materials and Methods section for a thorough derivation of the mathematical model. The tumor dynamics depend on $\alpha \sigma^{-1}$, a nondimensional number that represents the tissue stiffness effectively perceived by the tumor cells. For a fixed value of the tissue stiffness α, high values of σ (i.e., low mechanosensitivity) translate into poor cellular mechanical response. On the other hand, for low values of σ, tumor cells respond more intensely to mechanical compression. In the following, we will refer to $\alpha \sigma^{-1}$ as the effective stiffness of the tissue, and quantify its effect on tumor growth.

**Figure 4:**
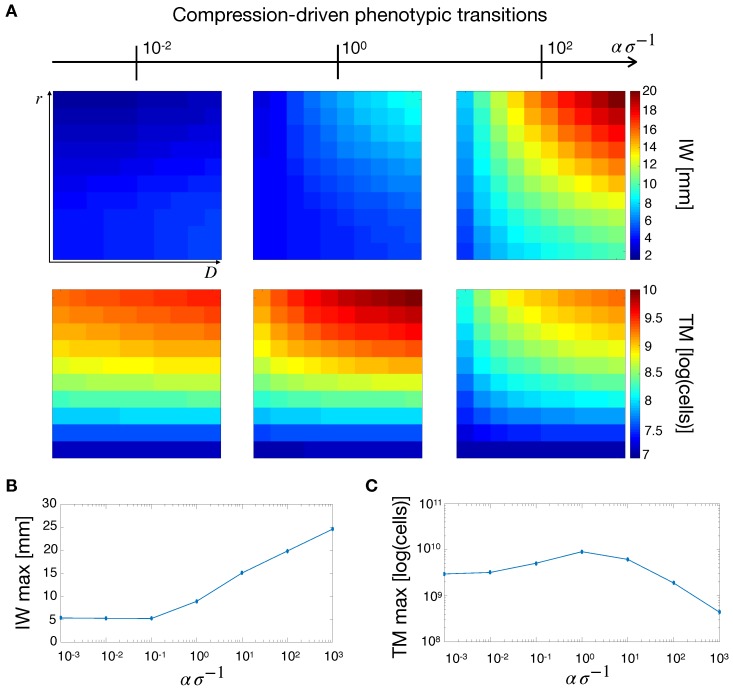
Tumor observables for mechanical-induced phenotypic switching. (**A**) Simulation maps of tumor IW and TM with respect to different values of effective stiffness $\alpha \sigma^{-1}$. From left to right $\sigma =10^{5},10^{3},10^{1}\mathrm{Pa}$ with $\alpha =10^{3}\mathrm{Pa}$. IW increases for increasing values of the α/σ ratio, whereas TM shows a maximum for intermediate values of effective stiffness. The maximum IW (**B**) and TM (**C**) are obtained for different values of effective stiffness, with $\alpha =10^{3}\mathrm{Pa}$, and σ varied. While IW shows an increasing trend with effective stiffness, TM displays an optimum for intermediate $\alpha \sigma^{-1}$ values.

Figure 4A shows simulation maps of tumor IW and TM for different values of the effective stiffness. For increasing values of $\alpha \sigma^{-1}$, IW increased especially for highly-diffusive tumors. On the other hand, TM displays a non-monotonic trend for increasing effective stiffness. Indeed, we observed a maximum for this quantity at intermediate values of $\alpha \sigma^{-1}$. This finding is highlighted in Figure 4B,C where the maximum values of IW and TM are displayed for different α/σ ratios. Figure 4B shows that for low effective stiffness IW is short. However, for intermediate values of $\alpha \sigma^{-1}$, IW increased linearly with increasing effective stiffness. Moreover, for low values of $\alpha \sigma^{-1}$, tumor cells are not responsive to mechanical stresses and the tumor does not acquire an infiltrative behavior. On the other hand, for increasing values of $\alpha \sigma^{-1}$, the transition from proliferative to migratory phenotype is enhanced, leading to tumors with more diffusive fronts. The situation changes with respect to TM, where we observe a maximum for TM at intermediate values of $\alpha \sigma^{-1}$, see Figure 4C. This occurs because at low effective stiffness tumors are compact and their cellular density tends to the carrying capacity of the tissue, thus limiting the overall tumor growth. In the case of high $\alpha \sigma^{-1}$ values, tumor cells proliferate less and acquire instead an invasive phenotype, which reduces tumor burden.

### 2.4. Nutrient-Driven Phenotypic Transitions

Now we consider transitions between the two cell phenotypes driven only by nutrient availability in the tumor microenvironment. In this case, a critical role was played by the vascular response to mechanical stresses. If mechanical pressure overcomes a critical threshold blood vessels collapse and nutrients are not delivered to the tumor.

**Figure 5:**
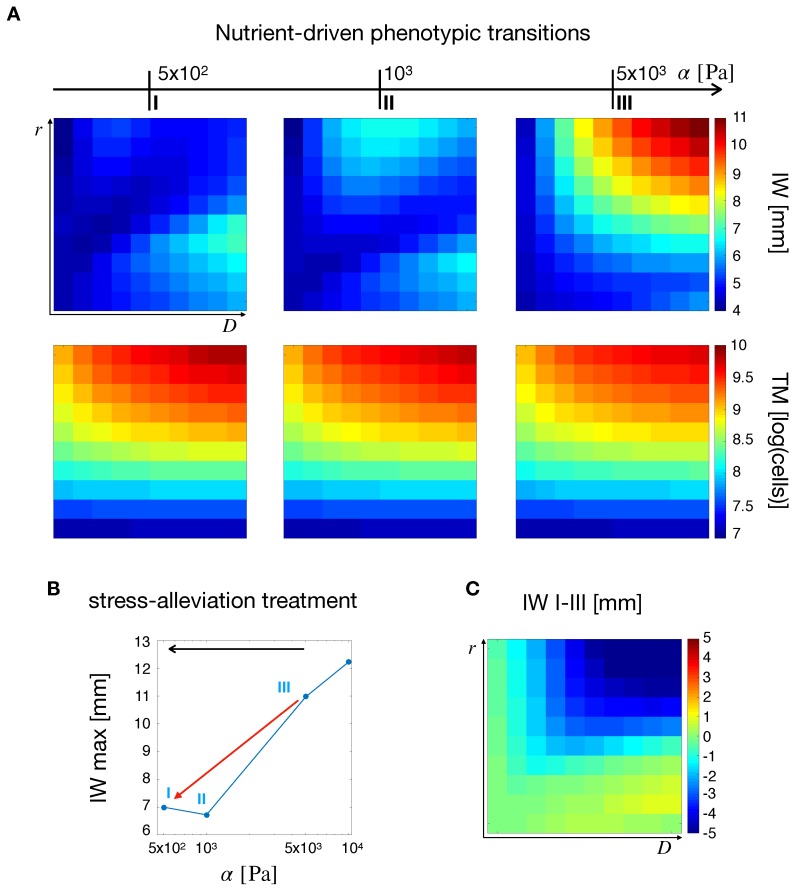
Tumor observables and the effects of a stress-alleviation therapeutic strategy in the case of nutrient-induced phenotypic switching. (**A**) From left to right, simulation maps of tumor IW and TM for varying tissue stiffness from $5\times 10^{2}\;\mathrm{Pa}$ to $5\times 10^{3}\;\mathrm{Pa}$. A non-trivial effect of stiffness on IW and a monotonic decrease in TM for increasing values of α is observed. (**B**) Maximum value of IW for different tumor stiffness values. The red arrow points in the direction of a stress-alleviation treatment, where shorter IWs are obtained for decreasing values of α. (**C**) Simulation map for the difference in IW between the points I and III in B. Gliomas with the highest values of *D* and *r* display the highest reductions.

Simulation maps in Figure 5A show the influence of tissue stiffness α on IW and TM. As α increases due to tumor growth, intratumoral mechanical stresses are exacerbated. Thus, for higher tissue compliances (Figure 5A, left column), the overall IW map displayed low values, with two peaks in the regions of low and high D. A similar behavior was observed for intermediate stiffness (Figure 5A, center column) where the peaks appeared lightly shifted towards the region of higher D. Finally, for high stiffness (Figure 5A, right column) IW displayed a unique peak in the top-right corner, i.e., at high *D* and *r*. Indeed, when the stiffness is low and vascular occlusion is not engaged, the highest values of IW are observed in two extremes, for highly proliferating and highly diffusive tumors. Tumors with high values of *D* and *r* fell in the middle, with intermediate values of IW. The situation changes as soon as the vasculature collapses due to higher stiffness (and stress) and nutrient deficiency pushes the cells towards a more migratory phenotype. In this case, tumors with the highest intrinsic motilities and proliferation rates developed longer infiltrative fronts, leading to the peak in IW at the top-right corner of the simulation map in Figure 5. The situation changes for TM, (second row of Figure 5A), where even if the increase in stiffness did not produce alterations in the pattern of TM in the (D,r) space, it leads to an overall tumor burden reduction. This observation was in line with the deficiency of nutrients that follows from vascular occlusion at higher stiffness. As the tumor became more deprived of nutrients, cell proliferation was reduced and TM decreased.

Figure 5B,C summarizes the effects of tissue stiffness on tumor IW. In particular, Figure 5B shows a decrease in the maximum IW for decreasing α. This can be understood as a stress-alleviation treatment, in which the tumor is treated with matrix degrading agents to decrease tissue stiffness. In fact, a decrease in stiffness, and thus in mechanical stress levels, may lead to less malignant tumor phenotypes in terms of a reduction in IW [26]. This is shown in Figure 5C, in which the difference between IW at $\alpha =5\times 10^{2}\;\mathrm{Pa}$ and $\alpha =5\times 10^{3}\;\mathrm{Pa}$ is displayed over the (D,r) space. The simulations also showed a decrease of IW in the top-right corner of Figure 5C. Even though the nutrient and vasculature spatial profiles do not dramatically change in these regions (see Figure S1), the improvement in tissue perfusion was able to reduce the tumor invasive behavior.

### 2.5. Chemo–Mechanically Induced Phenotypic Transitions

We now focus on the effects of both mechanical compression and nutrient deprivation on cellular phenotypic transitions. These are described by the ratio $t_{n}/t_{s}$, where $t_{n}$ and $t_{s}$ are the phenotypic transition rates related to nutrient availability and mechanical compression, respectively. For $t_{n}/t_{s}>1$ ($t_{n}/t_{s}<1$), cancer cells respond more (less) intensively to nutrient deprivation compared to mechanical stresses. We then simulate the effects of a stress-alleviation therapeutic strategy by decreasing the tissue stiffness from $\alpha =5\times 10^{3}\;\mathrm{Pa}$ to $\alpha =5\times 10^{2}\;\mathrm{Pa}$. In addition, two ranges of effective stiffness are considered, namely $\alpha \sigma^{-1}=\left[10^{-2},10^{-1}\right]$ and $\alpha \sigma^{-1}=\left[10^{1},10^{2}\right]$, accounting for different degrees of cell mechanosensitivity.

#### 2.5.1. Effects of Phenotypic Transitions on Tumor IW

**Figure 6:**
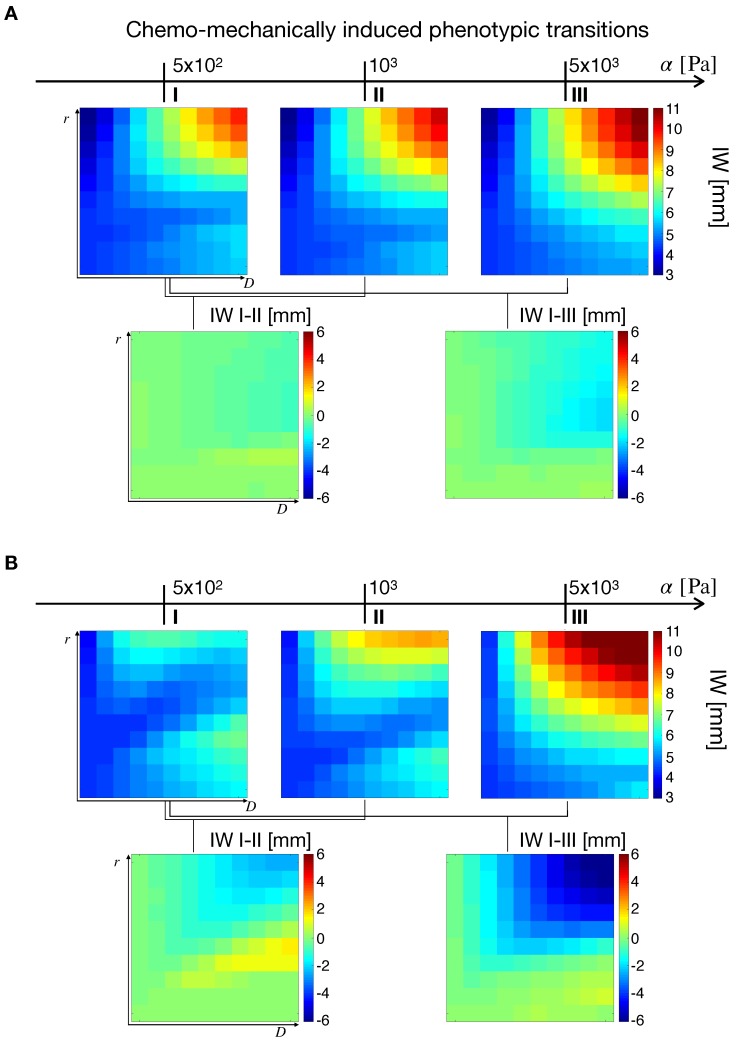
Simulation maps for the effects of chemo-mechanically induced transitions on tumor IW. In both cases (**A**,**B**), the top row shows three IW maps for different values of α, whereas the bottom row the IW variation occurring at different stiffness values. Simulations were obtained for $\alpha \sigma^{-1}=\left[10^{-2},10^{-1}\right]$ with $t_{n}/t_{s}=0.5$ (**A**) and $t_{n}/t_{s}=10$ (**B**).

We first analyze the effects of cellular responses to microenvironmental stimuli on the tumor IW. Figure 6A shows the simulation maps for $t_{n}/t_{s}=0.5$ and $\alpha \sigma^{-1}=\left[10^{-2},10^{-1}\right]$, i.e., in the case of mechanical response prevalent with respect to nutrient response and low mechanosensitivity. In the top row, simulations show higher IWs for increasing tissue stiffness, with the highest values at the top-right corner of the (D,r) space. The bottom row shows the effects of stress-alleviation treatments of different intensities, in which tissue stiffness is decreased from $\alpha =10^{3}\;\mathrm{Pa}$ to $\alpha =5\times 10^{2}\;\mathrm{Pa}$ (IW I–II, moderate intensity), and from $\alpha =5\times 10^{3}\;\mathrm{Pa}$ to $\alpha =5\times 10^{2}\;\mathrm{Pa}$ (IW I–III, high intensity). This results in only a slight variation of IW. Indeed, since in this case the cellular response to mechanical cues is stronger than for nutrients and the system is in the low mechanosensitivity regime, the effects of a stress reduction are limited on tumor IW.

Figure 6B deals with the case of nutrient response prevailing on the mechanical one, showing simulations with $t_{n}/t_{s}=10$ and $\alpha \sigma^{-1}=\left[10^{-2},10^{-1}\right]$, i.e., low mechanosentivity. Results displayed in Figure 6B recapitulate the observations in the case of nutrient-driven transitions (Figure 5; here it was $t_{s}=0$), with two peaks in the IW for low stiffness and a single maximum at high tissue rigidity. As the $t_{n}/t_{s}$ ratio takes large values it is reasonable to obtain similar results to the previous section (Figure 5), the mechanical response being quenched in the transition terms. Then, from the left map in the bottom row, two observations emerge. First, a slight decrease of IW in the top-right portion of the map was observed. This was a consequence of nutrient deprivation due to vascular occlusion, particularly occurring in highly infiltrative tumors (high *D* and *r* values). Second, a slight increase of IW in the region of D≫1 (as represented in Figure 3C), meaning that highly diffusive tumors benefited from a stress-alleviation strategy. The situation was different for the right simulation map in the bottom row of Figure 6B, referring to a more intense stress alleviation treatment (IW I–III). In this case, a decrease in IW for highly infiltrative gliomas was obtained, similar to what can be observed in Figure 5B,C. As before, highly infiltrative gliomas were more sensitive to changes in stiffness, and a reduction in mechanical compression results in a decrease of tumor IW.

The results for the IW difference at different $t_{n}/t_{s}$ ratios are summarized in Figure S2. Simulation results show that by increasing the nutrient-to-mechanical response ratio the IW decreases in highly invasive gliomas. This was particularly evident for the case of high intensity of stress-alleviation (displayed in the bottom row of Figure S2A). On the other hand, the top row shows a saddle in IW difference that emerges as $t_{n}/t_{s}$ increases. For high nutrient to stress response the model simulations qualitatively reproduce the results in [40]. In that work, a biphasic variation of IW for highly invasive gliomas for decreasing vaso-occlusion degrees was reported. In the case of low nutrient-to-mechanical response ratio, only slight variations of IW are observed for both intensities of the stress-alleviation strategy.

We perform the same analysis on nutrient-to-mechanical response in the case of high mechanosensitivity, i.e., $\alpha \sigma^{-1}=\left[10^{1},10^{2}\right]$. For brevity, we report the conclusive IW differences in the supplementary figures (Figures S2B, S3 and S4). For high $t_{n}/t_{s}$ ratios, the simulation maps resemble the results in Figure S2A, for low mechanosensitivity. Indeed, when the nutrient response overcame the mechanical one, the tumor behavior was driven by nutrient availability and a decrease in IW occurs for high intrinsic cellular diffusion and proliferation. On the other hand, for low nutrient-to-mechanical response, a reduction of IW for all tumors represented in the (D,r) space was observed. Since tumor cells were more sensitive to mechanical stimuli, a reduction in the stress level translates in a less migratory cell population, and thus in a reduction of infiltrative patterns.

#### 2.5.2. Effects of Phenotypic Transitions on TM

To conclude the study of chemo–mechanically induced phenotypic transitions, we analyzed the effects of nutrient-to-mechanical response and mechanosensitivity level on TM. For the sake of brevity, we report the difference plots for TM in the various cases in the Supplementary Figures (Figures S5–S7). Figure S5A shows the effects of phenotypic transitions on the variation of TM for different $t_{n}/t_{s}$ ratios at low cell mechanosensitivity. In this case, simulation maps show similar trends, with the intensity of the stress-alleviation treatment enhancing the variations in TM. For low $t_{n}/t_{s}$ ratios, a small decrease in tumor mass was observed at the top of the (D,r) space. This effect becomes less apparent when the nutrient response prevails over the mechanical one, see the simulation map for $t_{n}/t_{s}=10$. In this case an overall increase of TM was predicted for a significant part of the simulation map, with a particular increase for tumors with increased cellular motility and proliferation rate. Indeed, if $t_{n}/t_{s}<1$, a reduction in mechanical stress levels translated in a decrease of TM, as shown in Figure 4C for mechanical-driven transitions. Since cell mechanosensitivity was low, a reduction in the stress levels leads to dense tumors with a compact radius and a decreased TM. On the other hand, for high nutrient-to-mechanical response ratios, tumor evolution is significantly influenced by nutrient dynamics. When stress-alleviation strategies were pursued, vascular occlusion was reduced and nutrient supply was restored. This leads to tumors with higher densities and increased cellular proliferation.

To conclude the analysis for TM, the case of high mechanosensitivity, i.e., $\alpha \sigma^{-1}=\left[10^{1},10^{2}\right]$, was investigated (see Figure S5B). In this case, the treatment induced a significant increase of TM for low $t_{n}/t_{s}$ ratios. Indeed, a predominant mechanical response in highly mechanoresponsive cells shifted the population to a more proliferative state in a stress-alleviation treatment. Cells turned from being for the most part migratory to actively proliferating, which resulted in a significant increase in TM. The increase was more evident for cells with high intrinsic proliferation rates, which contribute the most to the overall tumor burden. As the $t_{n}/t_{s}$ ratio increases, this behavior is gradually lost and the solutions for nutrient-driven phenotypic transitions are partially recovered (see Figure 5A). For $t_{n}/t_{s}=10$, an increase of TM was observed for highly invasive gliomas, leading to tumors with reduced infiltrative patterns but increased cellular content.

#### 2.5.3. High Effective Stiffness and Low Nutrient to Stress Response Are the Best Fit for Stress-Alleviation Treatments

**Figure 7:**
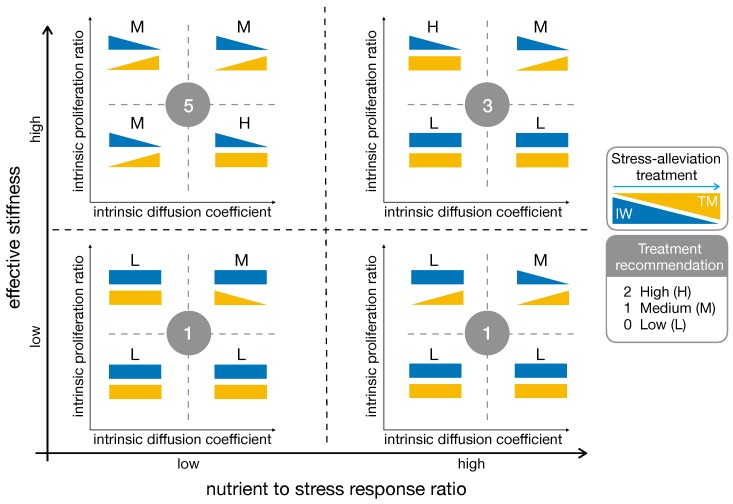
Summary of model results for chemo-mechanically induced phenotypic transitions. The triangles denote an increase (or decrease) of IW (blue) or TM (yellow) when a stress-alleviation treatment is performed. Rectangles denote a scarce effect of the treatment. The graph spans different levels of cell mechanosensitivity (vertical axis) and nutrient-to-mechanical response (horizontal axis). The labels and the circled numbers are related to the treatment recommendation score (L: low; M: medium; H: high), with higher numbers identifying better treatment outcomes.

Model results for the influence of stress-alleviation strategies on chemo–mechanically induced phenotypic transitions are summarized in Figure 7. In several cases the treatment produced opposing results: the IW decreases, but an increase in TM was observed at the same time. Indeed, if cells reduced their migratory phenotype due to the treatment, they become more proliferative, increasing TM. This may lead to tumors with high cellular densities but decreased invasive behaviors, possibly resulting in better outcomes for surgery. Another interesting point was visible in the bottom–left quadrant of the scheme, where the results for low mechanosensitivity and low nutrient-to-mechanical response are summarized. In this case the stress-alleviation strategy had limited impact on the tumor, being poorly effective in reducing its infiltrative behavior. For tumors in this area, adjuvant treatments potentially increasing cell mechanosensitivity may be sought, restoring the efficacy of the stress-alleviation strategy. To conclude, we estimate the impact of stress-alleviation strategies on the different cases by introducing a scoring system. We assign 0 to the cases in which treatment recommendation is low (L), i.e., the treatment does not produce significant changes or worsens tumor behavior. We assign 1 to the cases in which treatment recommendation is medium (M), e.g., reduction of IW together with increase of TM; and 2 for the cases of potentially successful outcomes (the treatment is highly recommended—H), i.e., reduction of IW and no change or decrease in TM. We report the labels of the different cases in Figure 7, and compute the resulting score for the fitness of the stress-alleviation treatment. The best fit to the treatment is obtained in the top-left quadrant, i.e., for low nutrient to stress response and high effective stiffness. On the other hand, low mechanosensitivity led to poor scores, discouraging the use of this treatment for those situations.

## 3. Discussion

We presented a mathematical model describing tumor growth and invasion, and we specialized it to the case of gliomas. The model is based on a system of partial differential equations, coupling tumor, nutrient and vasculature dynamics. We focus on the GoG mechanism, defining two cellular populations with mutually exclusive characteristics, i.e., migratory and proliferative cells. We extended the analysis in [40] by focusing on the combined impact of mechanical compression and nutrient availability on glioma progression. We analyzed the impact of microenvironmental stimuli, in terms of mechanical compression and nutrient availability, on glioma progression. In addition, we investigated the effects of cellular mechanosensitivity and nutrient-to-mechanical cellular response on two main tumor characteristics, namely the infiltration width (IW) of the tumor and its mass (TM).

Model simulations show that the combination of responses from external stimuli is able to generate a wide pattern of tumor behaviors. When the tumor microenvironment is modified—for example after a stress-alleviation treatment—tumors characterized by different response parameters react to environmental modifications in different ways. Notably, for low cellular mechanosensitivity and low nutrient-to-mechanical response, a stress-alleviation strategy is not able to effectively reduce tumor infiltrative behavior. On the contrary, mechanosensitive tumors reduce diffuse invasion after stress-alleviation. In addition, the model highlights the influence of intrinsic tumor features, i.e., cellular diffusivity *D* and proliferation rate *r*, on the intensity of treatment response. Indeed, if on the one hand for some intrinsic proliferation rates a change in stiffness does not result in a modification of TM or IW, for the other hand for higher values of cell proliferation the two variables change significantly. These findings highlight the presence of two distinct mechanisms related to tumor progression. For a given microenvironmental stimulus, cancer cells are characterized by their sensitivity [45,46] to the stimulus—described by the parameter σ in our model—and their response to it, i.e., the intensity of the phenotypic transition—described by $t_{n}$ and $t_{s}$. As the model suggests, there can be cases for which cell sensing is high, but cellular responses are low, and vice-versa. This translates in different tumor behaviors, modulated by the intrinsic cellular diffusivity and proliferation ratio. Interestingly, these two distinct mechanisms may be targets for future therapies. For example, the model predicts that by making cells more sensitive to mechanical stimuli [47], it is possible to elicit effective tumor responses to stress-alleviation treatments. Finally, model results highlight the existence of a trade-off between IW and TM for stress-alleviation treatments. If, on the one hand alleviation of mechanical stresses reduces tumor infiltrative tendency, on the other hand it may lead to tumors with an increased number of cells, intensifying the burden in the normal tissue. Based on model results, we notice that treatments based on the alleviation of mechanical stresses in the tissue might fail for some classes of tumors, due to the complexity of the mechanisms underlying tumor progression and patient heterogeneity (e.g., different cellular mechanosensitivities, as displayed in the Results section). Patient-based estimation of intrinsic tumor features, such as cell proliferation and migration rates, as well as mechanical sensitivity, would be fundamental for the design of tailored treatments for glioma patients. The experiments shown in Section 2.1 are a step forward in this direction, representing more a “motivation” for further studies, rather than a realistic quantification. We were not focused in modeling these experiments, rather in using them as a proof for the evidence of phenotypic responses to mechanical stimuli. Even though the glioma cell lines explored in the study were in vitro cultured, and we limited the investigation to a single compression level, similar assays could be applied to cells derived from cancer patients, and assuming multiple compression stresses and observation time points. In addition, culturing conditions could be varied (e.g., oxygen and glucose levels, growth factors etc.) to evaluate tumor response in environments that mimic more closely in vivo circumstances. Tumors are probably highly heterogeneous also from the mechanical-response point of view, with subpopulations behaving like H4 or A172 cells, or likely in even other fashions. The mechanical response of cancer cells could therefore be used as a potential mechanical biomarker, assisting clinicians in designing personalized therapeutic strategies. Regarding the different response to mechanical compression exhibited by the two cell lines in this study, an explanation may come from their origin. Gliomas can be classified into low grade or benign (grade I–III) and into high grade or malignant, such as glioblastoma (grade IV)—also termed Glioblastoma Multiforme (GBM). A common characteristic, and a major cause of the clinical symptoms seen in patients suffering from GBM, is the compression of surrounding healthy brain tissue by the primary tumor mass. The presence of compressive forces in the cranium is responsible for the midline shift, a typical characteristic of patients with GBM, which represents a negative prognostic factor [48]. A possible explanation for the different response of H4 and A172 to wound healing assays could be based on the fact that H4 cells are derived from a patient diagnosed with a low grade neuroglioma [49], whereas A172 cells derive from a GBM patient—thus from a high grade tumor [49]. The H4 cell line might be more sensitive to extracellular mechanical stimuli compared to the A172 line, which was pre-exposed to mechanical forces in vivo and might have become less sensitive to mechanical compression (due to adaptation).

We conclude our discussion by pointing out some limitations of the current approach, as well as some possible future developments. Our work focused on phenotypic plasticity in gliomas by virtue of the GoG mechanism (cell responds to nutrients and stress changes). In this context, we are not explicitly modeling intrinsic genetic heterogeneity in the tumor. Our model implicitly takes this feature into account through the definition of the intrinsic cellular motility and proliferation rate coefficients (*D* and *r*). The inter-tumoral differences are encoded in these parameters, which vary between different patients and are as well affected by tumor microenvironment. There have been different studies exemplifying the impact of intrinsic heterogeneity on tumor progression and particularly on therapy resistance (see for example [50,51]). However, the impact of heterogeneity due to phenotypic plasticity is not yet completely understood to which we intend to contribute with this work. As discussed in recent reviews [4,52], this modeling choice—although neglecting the specific form of the genetic heterogeneities in the tumor—is still albe to effectively describe gliomas and their responses to treatment. One of the main simplifications in the model is related to the description of mechanical stresses in the tumor. In our formulation, we take into account only compressive stress states and we correlate them to cellular density [53,54]. This approach is quite phenomenological, and for a more thorough account of mechanical issues models based on continuum mechanics and mixture theory should be employed—see [55,56,57,58,59,60,61] for some recent models, and [28,62] for in-depth reviews. Nonetheless, we believe that our findings are able to provide valuable insights into the response of gliomas to microenvironmental stimuli, and to suggest new therapeutic strategies. We implicitly take into account cell apoptosis as a reduction of the net proliferation rate in the logistic growth term. Although more specific cell death mechanisms could have been considered, such as necrosis, they would impact the transient dynamics rather the long-term behavior of the system [30,38,63]. Furthermore, we modeled the migration/proliferation dichotomy in the simplest possible way, focusing on phenotypic diversity driven by nutrient levels and mechanical stresses. More complete formulations could be considered, taking into account additional tumor-related factors. As another limitation of the current approach, we carried out simulations enforcing spherical symmetry of the problem. This was to focus on the effects of model dynamics rather than on geometrical influences deriving from realistic three-dimensional geometries. As a matter of fact, these geometric features could be readily included in the model from MRI data of actual patients.

Currently, we limited the discussion to chemical and mechanical effects on tumor cells, however it is known that other intrinsic and extrinsic factors play a role in tumor progression. We plan to extend the modeling framework to investigate the interactions between gliomas and immune cells, being aware of the potential benefits of cancer immunotherapies. The dynamics of immune cells could be coupled to microenvironmental stimuli, to give a more detailed picture of glioma growth and invasion. In addition to that, the model could be used as a framework to study combined therapies in gliomas. Indeed, a decrease of invasiveness correlated to an increased mass is predicted for some tumor types as a result of stress-alleviation strategies. The model could be extended to analyze the effects of chemotherapy or radiation combined to stress-alleviation, as a mechanism to reduce tumor burden and improve the chances of cure.

We strongly believe that novel investigations on the interactions between tumor microenvironment and cellular plasticity, informed by mathematical modeling, could allow for a better understanding of disease progression, with the final goal of aiding the design of effective therapeutic treatments.

## 4. Materials and Methods

### 4.1. Mathematical Model

We introduced the normalized density of proliferating and migrating glioma cells, denoted by $\rho_{p}$ and $\rho_{m}$, respectively. The system of equations describing the spatio-temporal evolution of the two latter quantities reads:

(2) $$\begin{array}{c} \frac{\partial \rho_{p}}{\partial t}=r\rho_{p}\left[1-\left(\rho_{p}+\rho_{m}\right)\right]-T_{\mathrm{pm}}\rho_{p}+T_{\mathrm{mp}}\rho_{m}, \end{array}$$

(3) $$\begin{array}{c} \frac{\partial \rho_{m}}{\partial t}=D\nabla^{2}\rho_{m}+T_{\mathrm{pm}}\rho_{p}-T_{\mathrm{mp}}\rho_{m}, \end{array}$$

in which $T_{\mathrm{pm}}$ and $T_{\mathrm{mp}}$ are the transition rates between the two cell phenotypes. The system in Equations (2) and (3) can be reduced to a single equation for the total density of glioma cells $\rho =\rho_{p}+\rho_{m}$ by considering that $T_{\mathrm{pm}}\rho_{p}=T_{\mathrm{mp}}\rho_{m}$. This is a plausible assumption, since the intracellular processes regulating the phenotypic switch operate at a shorter time scale than cell proliferation and migration [64]. Thus, we can express ρ as

(4) $$\rho =\rho_{p}\left(1+\frac{T_{\mathrm{pm}}}{T_{\mathrm{mp}}}\right)=\rho_{m}\left(1+\frac{T_{\mathrm{mp}}}{T_{\mathrm{pm}}}\right),$$

and $\rho_{p}$ and $\rho_{m}$ as

(5) $$\rho_{p}=\rho {\left(1+\frac{T_{\mathrm{pm}}}{T_{\mathrm{mp}}}\right)}^{-1},\;\rho_{m}=\rho {\left(1+\frac{T_{\mathrm{mp}}}{T_{\mathrm{pm}}}\right)}^{-1}.$$

Summing Equations (2) and (3), and substituting for $\rho_{p}$ and $\rho_{m}$ the expressions in Equation (5), the equation for the total density ρ reads:

(6) $$\frac{\partial \rho }{\partial t}=D\nabla^{2}\left(T_{m}\rho \right)+rT_{p}\rho \left(1-\rho \right),$$

in which the quantities $T_{m}$ and $T_{p}$ are defined as

(7) $$T_{m}=\frac{T_{\mathrm{pm}}}{T_{\mathrm{pm}}+T_{\mathrm{mp}}},\;T_{p}=\frac{T_{\mathrm{mp}}}{T_{\mathrm{pm}}+T_{\mathrm{mp}}}.$$

Now, we specify the equations for the normalized nutrient concentration and vascular density:

(8) $$\begin{array}{c} \frac{\partial n}{\partial t}=D_{n}\nabla^{2}n+p_{n}vS_{n}\left(1-n\right)-d_{n}\rho n, \end{array}$$

(9) $$\begin{array}{c} \frac{\partial v}{\partial t}=D_{v}\nabla^{2}v+p_{v}\left(1-n\right)v\left(v_{c}-v\right), \end{array}$$

in which $D_{n}$ and $D_{v}$ are the diffusion coefficient of the nutrient and vascular dispersal rate, respectively; $p_{n}$ is the supply rate of nutrient from the vasculature, $S_{n}$ is a function accounting for the collapse of blood vessels due to mechanical compression, $d_{n}$ is the nutrient consumption rate by tumor cells, $p_{v}$ is the generation rate of new blood vessels and $v_{c}$ is the carrying capacity of blood vessels in the tumor.

To close the problem, we defined suitable constitutive relations for the phenotypic transition terms, the mechanical stress sensed by tumor cells, and the vascular collapse term.

We assumed that phenotypic transitions of tumor cells were driven by both chemical and mechanical effects, and we define $T_{\mathrm{pm}}$ and $T_{\mathrm{mp}}$ as

(10) $$\begin{array}{c} T_{\mathrm{pm}}=t_{n}\left(\gamma_{n}-n\right)+t_{s}\frac{\Sigma +\gamma_{s}}{\Sigma +\sigma }, \end{array}$$

(11) $$\begin{array}{c} T_{\mathrm{mp}}=t_{n}n+t_{s}\left(1-\frac{\Sigma +\gamma_{s}}{\Sigma +\sigma }\right), \end{array}$$

in which $t_{n}$ and $t_{s}$ describe the transition rates due to nutrient and mechanical stress, respectively; Σ represents the mechanical compression sensed by tumor cells, and σ describes cell mechanosensitivity (i.e., for high values of σ, cells are more refractory to transitions due to mechanical stress, and viceversa). In addition, $\gamma_{n}$ and $\gamma_{s}$ are small regularization terms, accounting for possible transitions when *n* or Σ are zero. For these choices of $T_{\mathrm{pm}}$ and $T_{\mathrm{mp}}$, the transition terms $T_{m}$ and $T_{p}$ read

(12) $$\begin{array}{c} T_{m}=\frac{t_{n}}{\gamma_{n}t_{n}+t_{s}}\left(\gamma_{n}-n\right)+\frac{t_{s}}{\gamma_{n}t_{n}+t_{s}}\frac{\Sigma +\gamma_{s}}{\Sigma +\sigma }, \end{array}$$

(13) $$\begin{array}{c} T_{p}=\frac{t_{n}}{\gamma_{n}t_{n}+t_{s}}n+\frac{t_{s}}{\gamma_{n}t_{n}+t_{s}}\left(1-\frac{\Sigma +\gamma_{s}}{\Sigma +\sigma }\right). \end{array}$$

To define an expression for Σ we accounted for mechanical compression of the cells in a phenomenological way, as previously done for the example in [46,53,65]. In particular, we assume that tumor cells feel mechanical compression for increasing densities. Thus, we consider that compression is a monotonic increasing function of tumor cell density, and tends to infinity at ρ=1, i.e., close to the carrying capacity. This relationship is modeled by the following expression:

(14) $$\Sigma =\alpha \frac{\rho }{1-\rho },$$

in which α is a constant accounting for the stiffness of the tissue.

Finally, for $S_{n}$ in (8), we assume that there exists a critical mechanical compression level $\Sigma_{\mathrm{cr}}$ above which vascular functionality is compromised [42]. Since this parameter was not available in literature for gliomas, we estimated it to be of the same order of magnitude as the brain tissue stiffness. The assumed functional form accounting for this behavior is

(15) $$S_{n}=H\left(\Sigma -\Sigma_{\mathrm{cr}}\right),$$

in which H is a smooth approximation of the decreasing step function, equal to one for Σ≈0 Pa and equal to zero for $\Sigma \gg \Sigma_{\mathrm{cr}}$.

Without loss of generality, we enforced spherical symmetry in the model equations to investigate the effects of parameter changes on a simple geometry. The spherically symmetric formulation of the problem is reported in Appendix A, whereas we summarize the parameter values used in the simulations in Table 1.

### 4.2. Experimental Procedure

#### 4.2.1. Cell Culture

The human glioma cell lines H4 and A172 were obtained from the American Type Culture Collection (ATCC, Manassas, VA, USA) and were maintained in Dulbecco’s modified eagle’s medium (DMEM) supplemented with 10% fetal bovine serum (FBS) and 1% antibiotics. Both cell lines were incubated at 37 ${}^{{}^{\circ}}$C and 5% ${\mathrm{CO}}_{2}$ in a humidified incubator.

#### 4.2.2. Application of Mechanical Compression

For the generation of solid stress in cancer cells, the transmembrane pressure device was employed as previously described [70]. Briefly, cancer cells were seeded in the inner chamber of a transwell insert (Greiner Bio-one, Frickenhausen, Germany), and a 1.5 mm-thick agarose gel was added on top of the cells preventing any contact between piston and cells, providing a uniform distribution of the applied force. A piston of a predefined weight was placed on the top of the agarose gel and the cells were exposed to 4 mmHg stress. Control cells were covered with an agarose gel.

#### 4.2.3. Alamar Blue Assay

Cancer cells were seeded in the inner chamber of a transwell insert ($10^{5}$ cells/insert) overnight. Alamar blue (AB) reagent (Thermo Fisher Scientific, Waltham, MA, USA) was subsequently added in culture medium (10% *v*/*v*) before- and after-compression and absorbance was measured at 570/600 nm after two hours of incubation. According to the manufacturer’s instructions, we created a calibration curve for each cell line in order to derive a correlation between the AB absorbance and the cell number (Supplementary Figure S8). Based on the derived calibration curves the AB absorbance was converted to the number of cells. Control cells were covered with an agarose cushion only.

#### 4.2.4. Wound Closure Assay

A wound healing assay was performed on cancer cells as previously described [70]. In brief, cancer cells were seeded in the inner chamber of a transwell insert ($2\times 10^{4}$ cells/insert) and allowed to form a monolayer. A scratch wound was then introduced on the cell monolayer, cells were washed twice with PBS and then were stimulated with 4 mmHg stress for 16 h. Control cells were covered with an agarose gel (i.e., 0 mmHg). Images from at least three different fields from each condition were taken at 0 h and 16 h. The cell-free area was quantified using the ImageJ software [71]. Quantification was performed for each condition using the following formula: (Area of the wound at 0 h − Area of the wound at 16 h)/(Area of the wound at 0 h).

## 5. Conclusions

In this work we investigated the influence of mechanical stresses on glioma progression. We analyzed the effects of mechanical compression on cellular migration and proliferation through a mathematical model, studying the impact of chemo-mechanical stimuli on glioma cell phenotypic transitions. We used the model to test the effects of cellular intrinsic parameters on stress-alleviation treatments, i.e., therapies that propose to reduce intratumoral mechanical stresses in order to improve tumor perfusion. We find that for some tumor classes stress-alleviation treatments reduce tumor infiltration but increase tumor mass. On the other hand, for other glioma types, stress-alleviation approaches provide negligible benefit to patients, suggesting the use of combined therapies to improve treatment outcomes.

## Figures and Tables

**Table 1:** Model parameters used in the simulations.

Parameter	Description	Value	Reference
*D*	Intrinsic diffusion coefficient of tumor cells	$2.73\times [10^{-3},10^{-1}]$ ${\mathrm{mm}}^{2}d^{-1}$	[38]
*r*	Intrinsic proliferation rate of tumor cells	$2.73\times [10^{-4},10^{-2}]$ $d^{-1}$	[38]
$\rho_{c}$	Carrying density of glioma cells	$10^{6}\;\mathrm{cells}\;{\mathrm{mm}}^{-3}$	[66]
$D_{n}$	Diffusion coefficient of nutrient	$1.51\times 10^{2}$ ${\mathrm{mm}}^{2}d^{-1}$	[67]
$p_{n}$	Nutrient supply rate from the vasculature	10 $d^{-1}$	[44]
$d_{n}$	Nutrient consumption by tumor cells	$5.73\times 10^{1}$ $d^{-1}$	[41]
$D_{v}$	Vasculature dispersal rate	$5\times 10^{-4}$ ${\mathrm{mm}}^{2}d^{-1}$	[68]
$p_{v}$	Vasculature formation rate	$10^{-1}$ $d^{-1}$	[69]
$v_{c}$	Vasculature carrying capacity	3	[42]
$\Sigma_{\mathrm{cr}}$	Critical stress for vasculature collapse	$10^{2}$ Pa	estimated
$\gamma_{n}$	Regularization term in (10)	1.01	model specific
$\gamma_{s}$	Regularization term in (11)	$10^{-2}\sigma$ Pa	model specific

**Figure A1:**
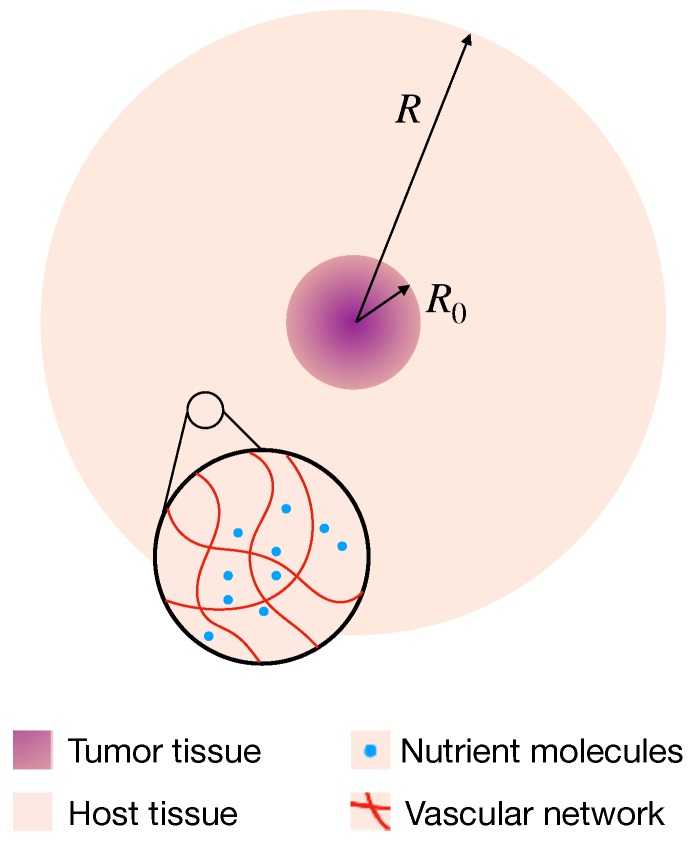
Schematics of the mathematical model. The tumor is placed at the center of the surrounding tissue, and the inlet highlights the presence of the vascular network and nutrient molecules in the system. $R_{0}$ and *R* denote the initial tumor radius and the computational domain, respectively.

## Supplementary Materials

The following are available online at https://www.mdpi.com/2072-6694/11/5/716/s1, Figure S1: Changes in nutrient concentration and vasculature density after a stress-alleviation treatment, Figure S2: Simulation maps displaying the impact of chemo-mechanically induced transitions on tumor IW at different nutrient to stress response ratios, Figure S3: Simulation maps displaying the effects of chemo-mechanically induced transitions on tumor IW at high mechanosensitivity and low nutrient to stress response ratio, Figure S4: Simulation maps displaying the effects of chemo-mechanically induced transitions on tumor IW at high mechanosensitivity and high nutrient to stress ratio, Figure S5: Simulation maps displaying the impact of chemo-mechanically induced transitions on TM, Figure S6: Simulation maps displaying the effects of chemo-mechanically induced transitions on TM at low mechanosensitivity, Figure S7: Simulation maps displaying the effects of chemo-mechanically induced transitions on TM at high mechanosensitivity, Figure S8: Calibration curves for the H4 and A172 cell lines in the Alamar Blue assay.

## Appendix A

The final system of equations in spherical symmetry reads:

(A1) $$\begin{array}{c} \frac{\partial \rho }{\partial t}-\frac{1}{x^{2}}\frac{\partial }{\partial x}\left[x^{2}D\frac{\partial }{\partial x}\left(T_{m}\rho \right)\right]-rT_{p}\rho \left(1-\rho \right)=0, \end{array}$$

(A2) $$\begin{array}{c} \frac{\partial n}{\partial t}-\frac{1}{x^{2}}\frac{\partial }{\partial x}\left(x^{2}D_{n}\frac{\partial n}{\partial x}\right)-p_{n}vS_{n}\left(1-n\right)+d_{n}\rho n=0, \end{array}$$

(A3) $$\begin{array}{c} \frac{\partial v}{\partial t}-\frac{1}{x^{2}}\frac{\partial }{\partial x}\left(x^{2}D_{v}\frac{\partial v}{\partial x}\right)-p_{v}\left(1-n\right)v\left(v_{c}-v\right)=0. \end{array}$$

in which *x* is the radial coordinate. To close the problem, we define the following boundary and initial conditions:

(A4) $$\begin{array}{c} \frac{\partial \rho }{\partial x}\left(0,t\right)=0,\;\frac{\partial n}{\partial x}\left(0,t\right)=0,\;\frac{\partial v}{\partial x}\left(0,t\right)=0, \end{array}$$

(A5) $$\begin{array}{c} \frac{\partial \rho }{\partial x}\left(R,t\right)=0,\;n\left(R,t\right)=1,\;v\left(R,t\right)=1, \end{array}$$

(A6) $$\begin{array}{c} \rho \left(x,0\right)=\rho_{0}\exp\left(-\frac{x^{2}}{2R_{0}^{2}}\right),\;n\left(x,0\right)=1,\;v\left(x,0\right)=1, \end{array}$$

in which *R* is the external radius of the domain, $\rho_{0}$ is the initial tumor density and $R_{0}$ is the initial radius of the tumor. A schematic of the model is shown in Figure A1.

Regarding the model parameters, these are taken from published data whenever available, or estimated by suitable physical and biological arguments. Table 1 summarizes the parameter values, together with their units and reference sources. We simulate the growth of a tumor over 1 year, and consider the following parameter values: $R=100\;\mathrm{mm},\;R_{0}=2\;\mathrm{mm},\;\rho_{0}=0.1$.

Once the mathematical model is parametrized, we proceed to its spatio-temporal discretization. This is performed via the finite element method, using linear elements; for time discretization, we use a standard backward Euler scheme, an implicit method. Once the equations are discretized, the model is implemented using the open source software FEniCS [72].

## References

[1] Sanai N., Alvarez-Buylla A., Berger M.S. (2005). Neural stem cells and the origin of gliomas. N. Engl. J. Med..

[2] Goodenberger M.L., Jenkins R.B. (2012). Genetics of adult glioma. Cancer Genet..

[3] Louis D.N., Perry A., Reifenberger G., Von Deimling A., Figarella-Branger D., Cavenee W.K., Ohgaki H., Wiestler O.D., Kleihues P., Ellison D.W. (2016). The 2016 World Health Organization classification of tumors of the central nervous system: A summary. Acta Neuropathol..

[4] Alfonso J., Talkenberger K., Seifert M., Klink B., Hawkins-Daarud A., Swanson K., Hatzikirou H., Deutsch A. (2017). The biology and mathematical modelling of glioma invasion: A review. J. R. Soc. Interface.

[5] Giese A., Bjerkvig R., Berens M., Westphal M. (2003). Cost of migration: Invasion of malignant gliomas and implications for treatment. J. Clin. Oncol..

[6] Albini A., Sporn M.B. (2007). The tumour microenvironment as a target for chemoprevention. Nat. Rev. Cancer.

[7] Quail D.F., Joyce J.A. (2013). Microenvironmental regulation of tumor progression and metastasis. Nat. Med..

[8] Northcott J.M., Dean I.S., Mouw J.K., Weaver V.M. (2018). Feeling Stress: The Mechanics of Cancer Progression and Aggression. Front. Cell Dev. Biol..

[9] Kalli M., Stylianopoulos T. (2018). Defining the role of solid stress and matrix stiffness in cancer cell proliferation and metastasis. Front. Oncol..

[10] Mascheroni P., Boso D., Preziosi L., Schrefler B.A. (2017). Evaluating the influence of mechanical stress on anticancer treatments through a multiphase porous media model. J. Theor. Biol..

[11] Muz B., de la Puente P., Azab F., Azab A.K. (2015). The role of hypoxia in cancer progression, angiogenesis, metastasis, and resistance to therapy. Hypoxia.

[12] Stylianopoulos T., Martin J.D., Chauhan V.P., Jain S.R., Diop-Frimpong B., Bardeesy N., Smith B.L., Ferrone C.R., Hornicek F.J., Boucher Y. (2012). Causes, consequences, and remedies for growth-induced solid stress in murine and human tumors. Proc. Natl. Acad. Sci. USA.

[13] Nia H.T., Liu H., Seano G., Datta M., Jones D., Rahbari N., Incio J., Chauhan V.P., Jung K., Martin J.D. (2017). Solid stress and elastic energy as measures of tumour mechanopathology. Nat. Biomed. Eng..

[14] Helmlinger G., Netti P.A., Lichtenbeld H.C., Melder R.J., Jain R.K. (1997). Solid stress inhibits the growth of multicellular tumor spheroids. Nat. Biotechnol..

[15] Janet M.T., Cheng G., Tyrrell J.A., Wilcox-Adelman S.A., Boucher Y., Jain R.K., Munn L.L. (2012). Mechanical compression drives cancer cells toward invasive phenotype. Proc. Natl. Acad. Sci. USA.

[16] Chen Q., Yang D., Zong H., Zhu L., Wang L., Wang X., Zhu X., Song X., Wang J. (2017). Growth-induced stress enhances epithelial-mesenchymal transition induced by IL-6 in clear cell renal cell carcinoma via the Akt/GSK-3*β*/*β*-catenin signaling pathway. Oncogenesis.

[17] Giese A., Loo M.A., Tran N., Haskett D., Coons S.W., Berens M.E. (1996). Dichotomy of astrocytoma migration and proliferation. Int. J. Cancer.

[18] Athale C., Mansury Y., Deisboeck T.S. (2005). Simulating the impact of a molecular ’decision-process’ on cellular phenotype and multicellular patterns in brain tumors. J. Theor. Biol..

[19] Hoek K.S., Eichhoff O.M., Schlegel N.C., Döbbeling U., Kobert N., Schaerer L., Hemmi S., Dummer R. (2008). In vivo switching of human melanoma cells between proliferative and invasive states. Cancer Res..

[20] Böttger K., Hatzikirou H., Chauviere A., Deutsch A. (2012). Investigation of the migration/proliferation dichotomy and its impact on avascular glioma invasion. Math. Model. Nat. Pheno..

[21] Hatzikirou H., Basanta D., Simon M., Schaller K., Deutsch A. (2012). ‘Go or grow’: The key to the emergence of invasion in tumour progression?. IMA J. Math. Appl. Med. Biol..

[22] Xie Q., Mittal S., Berens M.E. (2014). Targeting adaptive glioblastoma: An overview of proliferation and invasion. Neuro Oncol..

[23] Joyce J.A. (2005). Therapeutic targeting of the tumor microenvironment. Cancer Cell.

[24] Stylianopoulos T., Munn L.L., Jain R.K. (2018). Reengineering the Physical Microenvironment of Tumors to Improve Drug Delivery and Efficacy: From Mathematical Modeling to Bench to Bedside. Trends Cancer.

[25] Chauhan V.P., Martin J.D., Liu H., Lacorre D.A., Jain S.R., Kozin S.V., Stylianopoulos T., Mousa A.S., Han X., Adstamongkonkul P. (2013). Angiotensin inhibition enhances drug delivery and potentiates chemotherapy by decompressing tumour blood vessels. Nat. Commun..

[26] Papageorgis P., Polydorou C., Mpekris F., Voutouri C., Agathokleous E., Kapnissi-Christodoulou C.P., Stylianopoulos T. (2017). Tranilast-induced stress alleviation in solid tumors improves the efficacy of chemo-and nanotherapeutics in a size-independent manner. Sci. Rep..

[27] Polydorou C., Mpekris F., Papageorgis P., Voutouri C., Stylianopoulos T. (2017). Pirfenidone normalizes the tumor microenvironment to improve chemotherapy. Oncotarget.

[28] Byrne H.M. (2010). Dissecting cancer through mathematics: From the cell to the animal model. Nat. Rev. Cancer.

[29] Altrock P.M., Liu L.L., Michor F. (2015). The mathematics of cancer: Integrating quantitative models. Nat. Rev. Cancer.

[30] Swanson K.R., Alvord E.C., Murray J. (2000). A quantitative model for differential motility of gliomas in grey and white matter. Cell Prolif..

[31] Jbabdi S., Mandonnet E., Duffau H., Capelle L., Swanson K.R., Pélégrini-Issac M., Guillevin R., Benali H. (2005). Simulation of anisotropic growth of low-grade gliomas using diffusion tensor imaging. Magn. Reson. Med..

[32] Clatz O., Sermesant M., Bondiau P.Y., Delingette H., Warfield S.K., Malandain G., Ayache N. (2005). Realistic simulation of the 3-D growth of brain tumors in MR images coupling diffusion with biomechanical deformation. IEEE T. Med. Imaging.

[33] Hogea C., Davatzikos C., Biros G. (2008). Brain–Tumor interaction biophysical models for medical image registration. SIAM J. Sci. Comput..

[34] Colombo M.C., Giverso C., Faggiano E., Boffano C., Acerbi F., Ciarletta P. (2015). Towards the personalized treatment of glioblastoma: Integrating patient-specific clinical data in a continuous mechanical model. PLoS ONE.

[35] Angeli S., Stylianopoulos T. (2016). Biphasic modeling of brain tumor biomechanics and response to radiation treatment. J. Biomech..

[36] Agosti A., Cattaneo C., Giverso C., Ambrosi D., Ciarletta P. (2018). A computational framework for the personalized clinical treatment of glioblastoma multiforme. J. Appl. Math. Mech..

[37] Hatzikirou H., Deutsch A., Schaller C., Simon M., Swanson K. (2005). Mathematical modelling of glioblastoma tumour development: A review. Math. Model. Methods Appl. Sci..

[38] Harpold H.L., Alvord E.C., Swanson K.R. (2007). The evolution of mathematical modeling of glioma proliferation and invasion. J. Neuropathol. Exp. Neurol..

[39] Martirosyan N.L., Rutter E.M., Ramey W.L., Kostelich E.J., Kuang Y., Preul M.C. (2015). Mathematically modeling the biological properties of gliomas: A review. Math. Biosci. Eng..

[40] Alfonso J., Köhn-Luque A., Stylianopoulos T., Feuerhake F., Deutsch A., Hatzikirou H. (2016). Why one-size-fits-all vaso-modulatory interventions fail to control glioma invasion: In silico insights. Sci. Rep..

[41] Grimes D.R., Kelly C., Bloch K., Partridge M. (2014). A method for estimating the oxygen consumption rate in multicellular tumour spheroids. J. R. Soc. Interface.

[42] Mpekris F., Angeli S., Pirentis A.P., Stylianopoulos T. (2015). Stress-mediated progression of solid tumors: Effect of mechanical stress on tissue oxygenation, cancer cell proliferation, and drug delivery. Biomech. Model. Mechanobiol..

[43] Carmeliet P., Jain R.K. (2000). Angiogenesis in cancer and other diseases. Nature.

[44] Kelly C.J., Brady M. (2006). A model to simulate tumour oxygenation and dynamic [18F]-Fmiso PET data. Phys. Med. Biol..

[45] Paszek M.J., Weaver V.M. (2004). The tension mounts: Mechanics meets morphogenesis and malignancy. J. Mammary Gland Biol. Neoplasia.

[46] Chaplain M.A., Graziano L., Preziosi L. (2006). Mathematical modelling of the loss of tissue compression responsiveness and its role in solid tumour development. IMA J. Math. Appl. Med. Biol..

[47] Wong S.Y., Ulrich T.A., Deleyrolle L.P., MacKay J.L., Lin J.M.G., Martuscello R.T., Jundi M.A., Reynolds B.A., Kumar S. (2015). Constitutive activation of myosin-dependent contractility sensitizes glioma tumor-initiating cells to mechanical inputs and reduces tissue invasion. Cancer Res..

[48] Seano G., Nia H.T., Emblem K.E., Datta M., Ren J., Krishnan S., Kloepper J., Pinho M.C., Ho W.W., Ghosh M. (2019). Solid stress in brain tumours causes neuronal loss and neurological dysfunction and can be reversed by lithium. Nat. Biomed. Eng..

[49] Cesselli D., Beltrami A.P., Pucer A., Bourkoula E., Ius T., Vindigni M., Skrap M., Beltrami C.A. (2013). Human Low-Grade Glioma Cultures. Diffuse Low-Grade Gliomas in Adults.

[50] Sottoriva A., Spiteri I., Piccirillo S.G., Touloumis A., Collins V.P., Marioni J.C., Curtis C., Watts C., Tavaré S. (2013). Intratumor heterogeneity in human glioblastoma reflects cancer evolutionary dynamics. Proc. Natl. Acad. Sci. USA.

[51] Andor N., Graham T.A., Jansen M., Xia L.C., Aktipis C.A., Petritsch C., Ji H.P., Maley C.C. (2016). Pan-cancer analysis of the extent and consequences of intratumor heterogeneity. Nat. Med..

[52] Jackson P.R., Juliano J., Hawkins-Daarud A., Rockne R.C., Swanson K.R. (2015). Patient-specific mathematical neuro-oncology: Using a simple proliferation and invasion tumor model to inform clinical practice. Bull. Math. Biol..

[53] Perthame B., Quirós F., Vázquez J.L. (2014). The Hele–Shaw asymptotics for mechanical models of tumor growth. Arch. Ration. Mech. Anal..

[54] Lorenzi T., Lorz A., Perthame B. (2017). On interfaces between cell populations with different mobilities. Kinet. Relat. Mod..

[55] Preziosi L., Ambrosi D., Verdier C. (2010). An elasto-visco-plastic model of cell aggregates. J. Theor. Biol..

[56] Ramírez-Torres A., Rodríguez-Ramos R., Merodio J., Bravo-Castillero J., Guinovart-Díaz R., Alfonso J.C.L. (2015). Action of body forces in tumor growth. Int. J. Eng. Sci..

[57] Ramírez-Torres A., Rodríguez-Ramos R., Merodio J., Bravo-Castillero J., Guinovart-Díaz R., Alfonso J.C.L. (2015). Mathematical modeling of anisotropic avascular tumor growth. Mech. Res. Commun..

[58] Mascheroni P., Stigliano C., Carfagna M., Boso D.P., Preziosi L., Decuzzi P., Schrefler B.A. (2016). Predicting the growth of glioblastoma multiforme spheroids using a multiphase porous media model. Biomech. Model. Mechanobiol..

[59] Ambrosi D., Pezzuto S., Riccobelli D., Stylianopoulos T., Ciarletta P. (2017). Solid tumors are poroelastic solids with a chemo-mechanical feedback on growth. J. Elast..

[60] Mpekris F., Voutouri C., Papageorgis P., Stylianopoulos T. (2018). Stress alleviation strategy in cancer treatment: Insights from a mathematical model. J. Appl. Math. Mech..

[61] Mascheroni P., Carfagna M., Grillo A., Boso D., Schrefler B. (2018). An avascular tumor growth model based on porous media mechanics and evolving natural states. Math. Mech. Solids.

[62] Preziosi L. (2003). Cancer Modelling and Simulation.

[63] Swanson K., Rostomily R., Alvord E. (2008). A mathematical modelling tool for predicting survival of individual patients following resection of glioblastoma: A proof of principle. Br. J. Cancer.

[64] Farin A., Suzuki S.O., Weiker M., Goldman J.E., Bruce J.N., Canoll P. (2006). Transplanted glioma cells migrate and proliferate on host brain vasculature: A dynamic analysis. Glia.

[65] Byrne H., Preziosi L. (2003). Modelling solid tumour growth using the theory of mixtures. IMA J. Math. Appl. Med. Biol..

[66] Stein S., Zhao R., Haeno H., Vivanco I., Michor F. (2018). Mathematical modeling identifies optimum lapatinib dosing schedules for the treatment of glioblastoma patients. PLoS Comput. Biol..

[67] Grote J., Süsskind R., Vaupel P. (1977). Oxygen diffusivity in tumor tissue (DS-carcinosarcoma) under temperature conditions within the range of 20–40 C. Pflügers Arch..

[68] Kouvroukoglou S., Dee K.C., Bizios R., McIntire L.V., Zygourakis K. (2000). Endothelial cell migration on surfaces modified with immobilized adhesive peptides. Biomaterials.

[69] Scianna M., Bell C., Preziosi L. (2013). A review of mathematical models for the formation of vascular networks. J. Theor. Biol..

[70] Kalli M., Papageorgis P., Gkretsi V., Stylianopoulos T. (2018). Solid stress facilitates fibroblasts activation to promote pancreatic cancer cell migration. Ann. Biomed. Eng..

[71] Rueden C.T., Schindelin J., Hiner M.C., DeZonia B.E., Walter A.E., Arena E.T., Eliceiri K.W. (2017). ImageJ2: ImageJ for the next generation of scientific image data. BMC Bioinform..

[72] Logg A., Mardal K.A., Wells G. (2012). Automated Solution of Differential Equations by the Finite Element Method: The FEniCS Book.

