# Supplementary material for: On the Impact of Chemo-Mechanically Induced Phenotypic Transitions in Gliomas
Source: Cancers (Basel). 2019 May 24;11(5):716. doi: 10.3390/cancers11050716 (PMC6562768; doi:10.3390/cancers11050716)
Supplement: Supplementary file 1 [file cancers-11-00716-s001.pdf]

Supplementary Materials

On the Impact of Chemo-Mechanically Induced Phenotypic Transitions in Gliomas

Pietro Mascheroni, Juan Carlos Lopez Alfonso, Maria Kalli, Triantafyllos Stylianopoulos, Michael Meyer-Hermann and Haralampos Hatzikirou

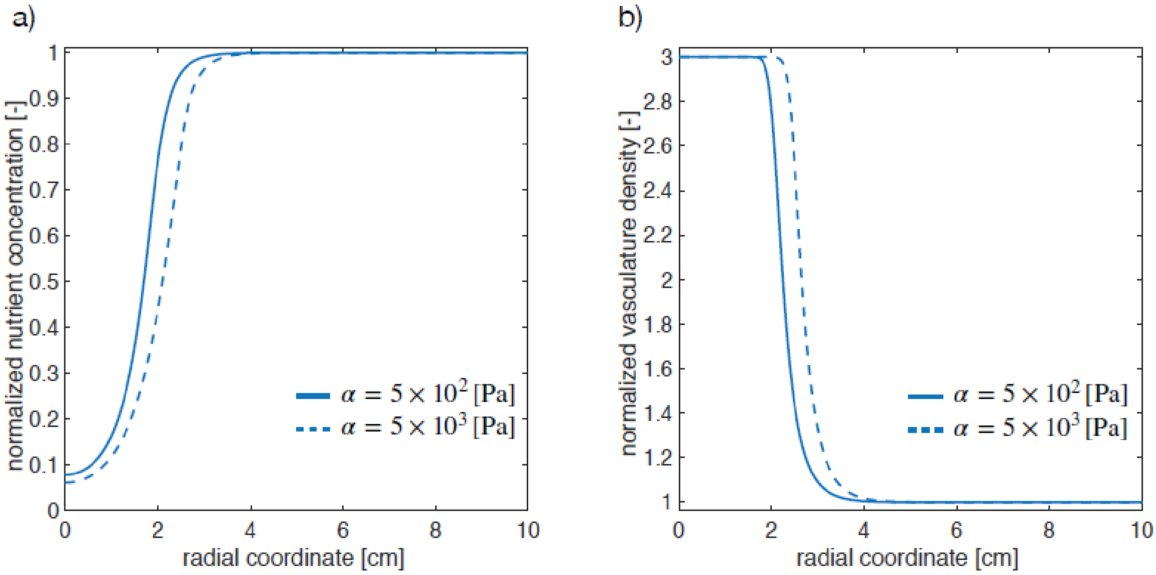

Figure S1. Changes in nutrient concentration (**a**) and vasculature density (**b**) after a stress-alleviation treatment (α is reduced from 5×10^3 Pa to 5×10^2 Pa). The simulations refer to the case of $D = 2.73 \times 10^{-1}$ mm^{2}d^{-1} and $r = 2.73 \times 10^{-2}$ d^{-1}.

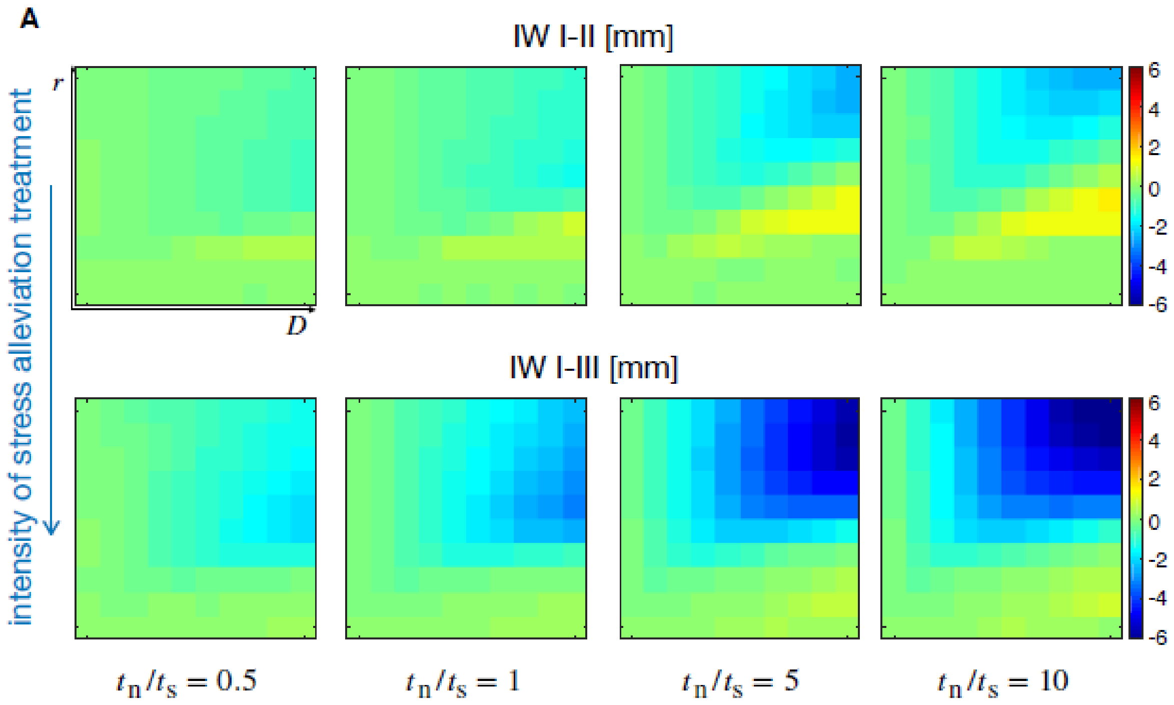

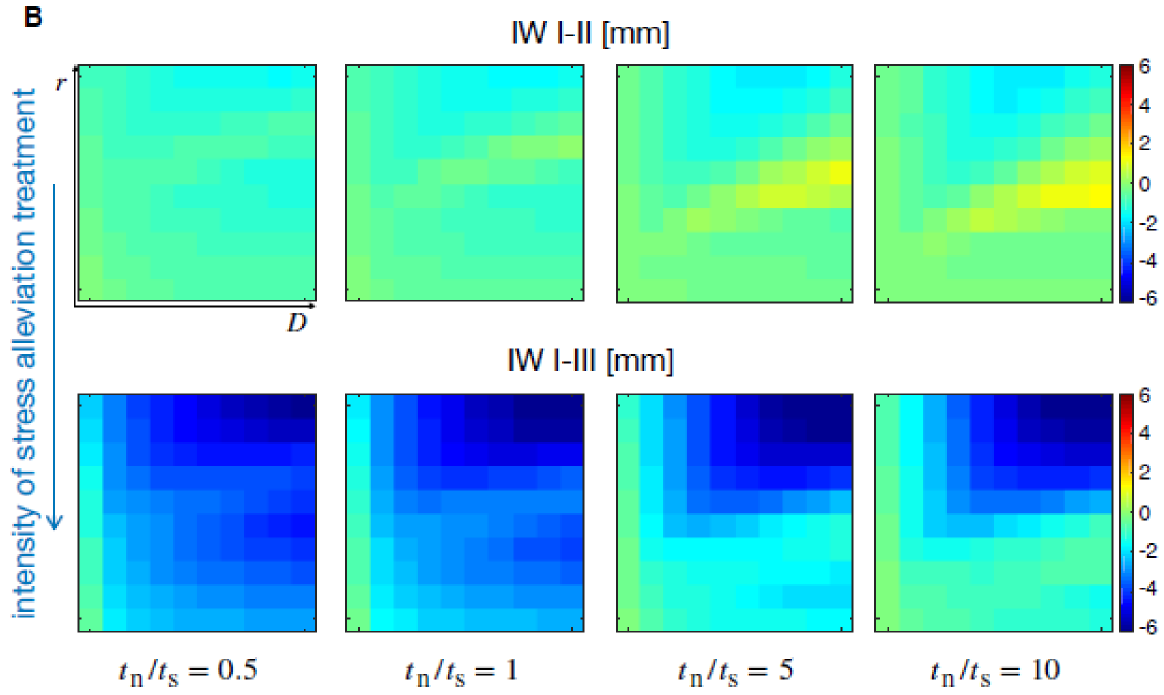

Figure S2. Simulation maps displaying the impact of chemo-mechanically induced transitions on tumor IW. In both cases (**A**,**B**), the top row shows the IW difference when tissue stiffness varies from $\alpha = 10^3$ Pa to $\alpha = 5 \times 10^2$ Pa, whereas the bottom row displays the IW variations for $\alpha = 5 \times 10^3$ Pa to $\alpha = 5 \times 10^2$ Pa. Simulations were obtained for low, i.e., $\alpha\sigma^{-1} = [10^{-2}, 10^{-1}]$ (**A**), and high, i.e., $\alpha\sigma^{-1} = [10^1, 10^2]$ (**B**) mechanosensitivity.

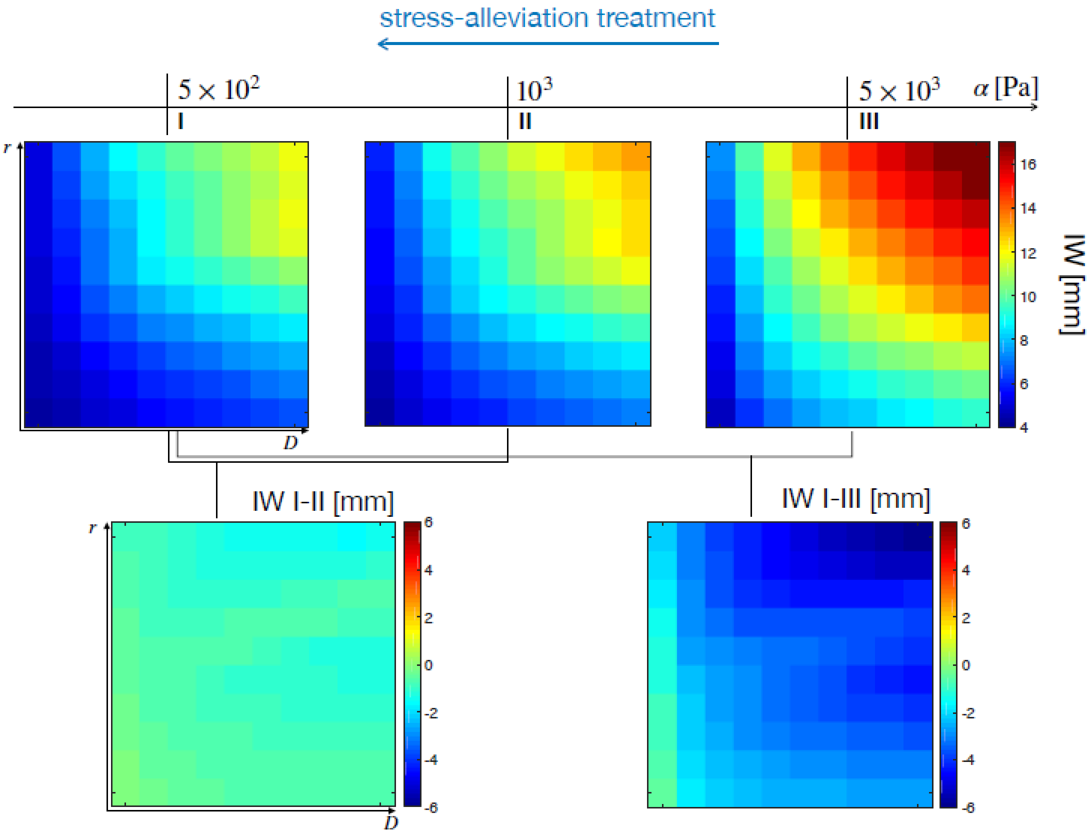

Figure S3. Simulation maps displaying the effects of chemo-mechanically induced transitions on tumor IW. The top row shows three IW maps for different values of α, whereas the bottom row displays the IW variation occurring at the different stiffness points. For these simulations, we used $t_n/t_s = 0.5$ and $\alpha\sigma^{-1} = [10^1, 10^2]$.

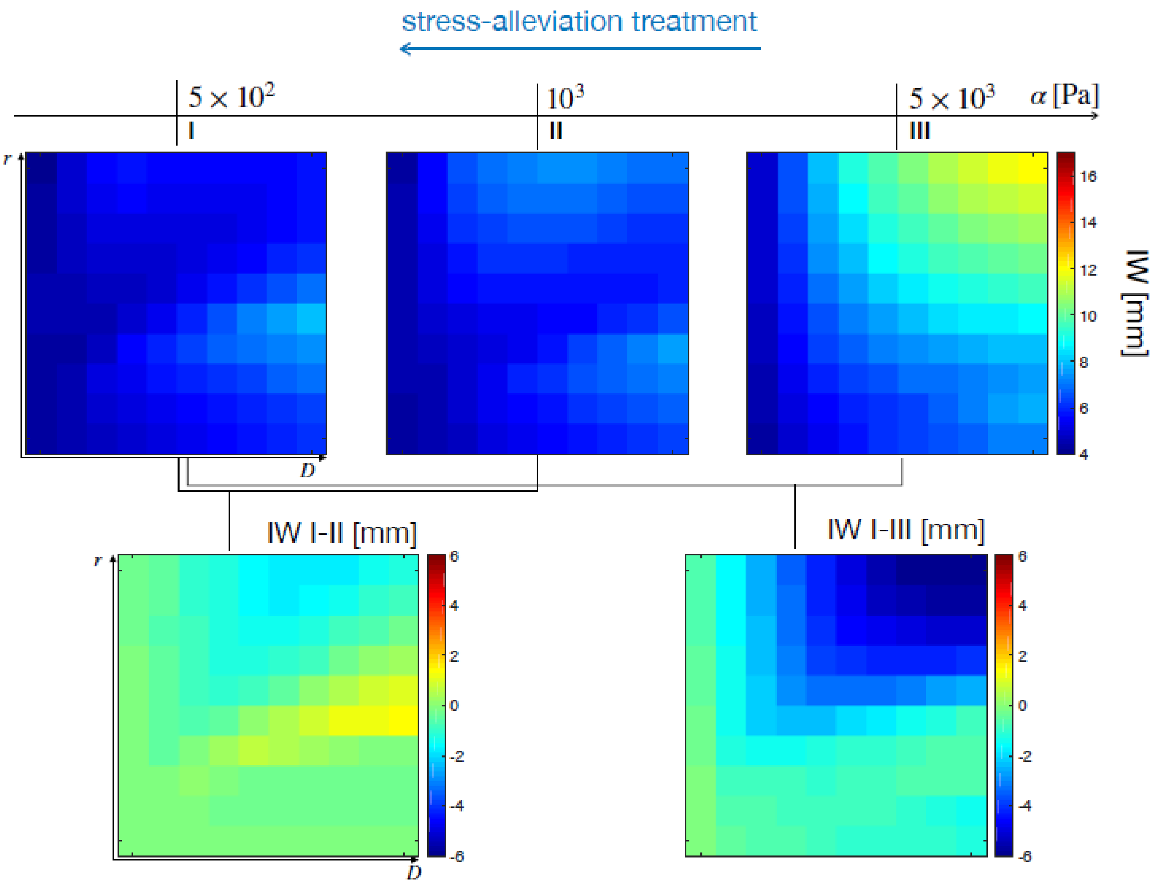

Figure S4. Simulation maps displaying the effects of chemo-mechanically induced transitions on tumor IW. The top row shows three IW maps for different values of α, whereas the bottom row displays the IW variation occurring at the different stiffness points. For these simulations, we used $t_n/t_s = 10$ and $\alpha\sigma^{-1} = [10^1, 10^2]$.

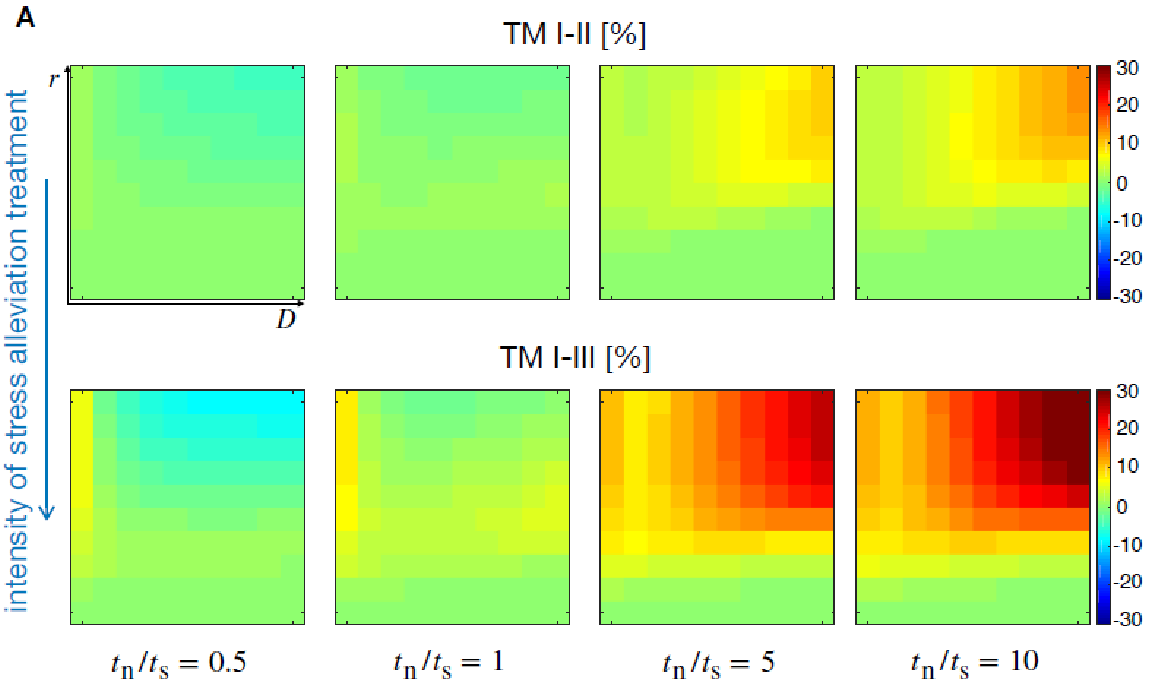

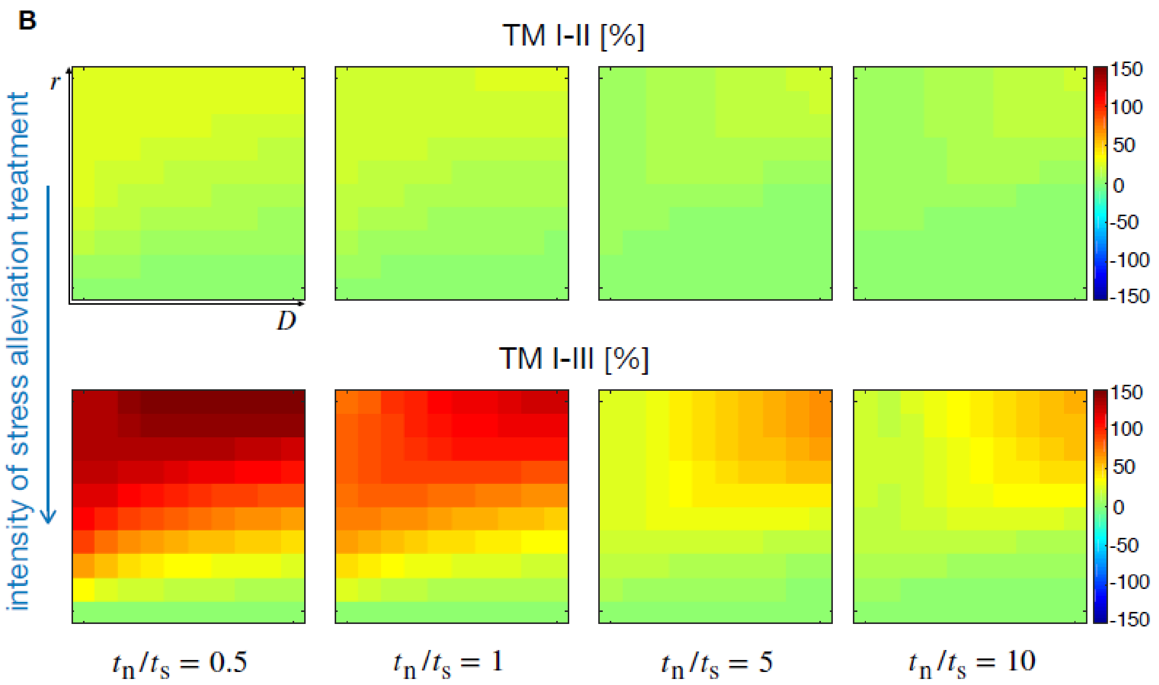

Figure S5. Simulation maps displaying the impact of chemo-mechanically induced transitions on TM. In both cases (**A**,**B**), the top row shows the TM difference for a reduction in tissue stiffness from $\alpha = 10^3$ Pa to $\alpha = 5 \times 10^2$ Pa, whereas the bottom row displays the TM variations for $\alpha = 5 \times 10^3$ Pa to $\alpha = 5 \times 10^2$ Pa. Simulations were obtained for low, i.e., $\alpha\sigma^{-1} = [10^{-2}, 10^{-1}]$ (**A**), and high, i.e., $\alpha\sigma^{-1} = [10^1, 10^2]$ (**B**) mechanosensitivity.

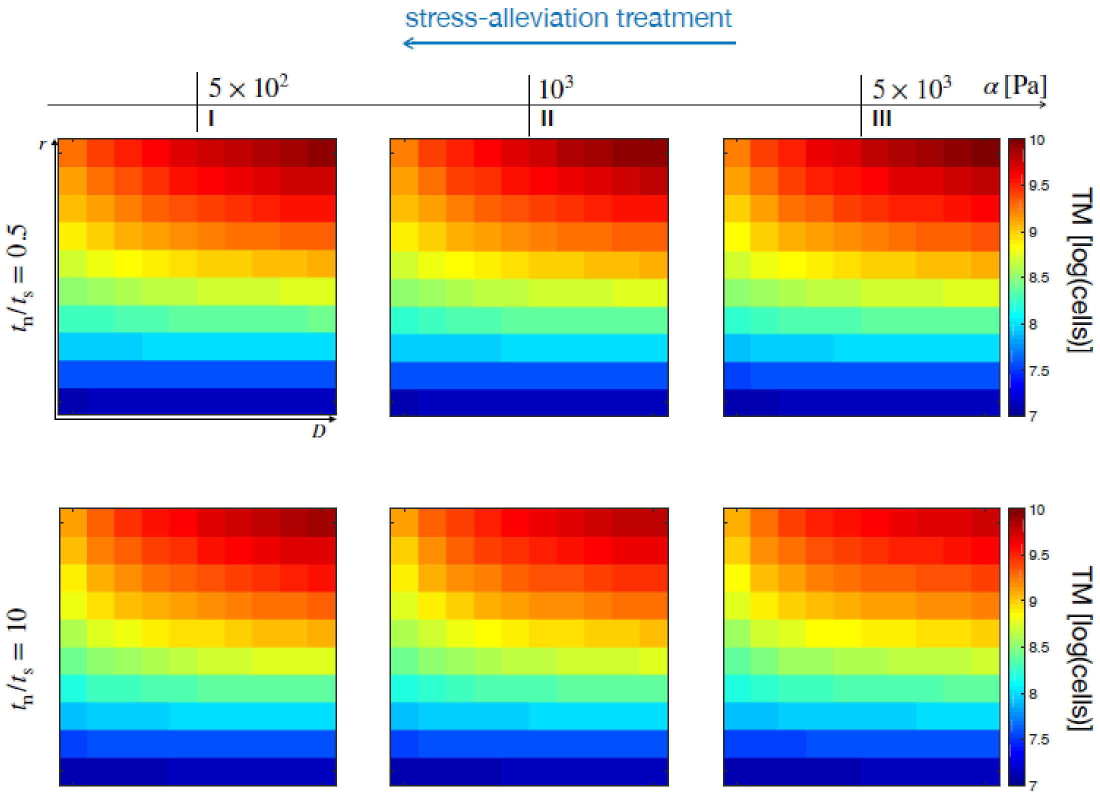

Figure S6. Simulation maps displaying the effects of chemo-mechanically induced transitions on TM. The top row shows three TM maps for different values of α at the ratio $t_n/t_s = 0.5$, whereas the bottom row displays TM values over the (D, r) space for the different stiffnesses at the $t_n/t_s = 10$ ratio. The simulations refer to the low mechanosensitivity case, i.e., $\alpha\sigma^{-1} = [10^{-2}, 10^{-1}]$.

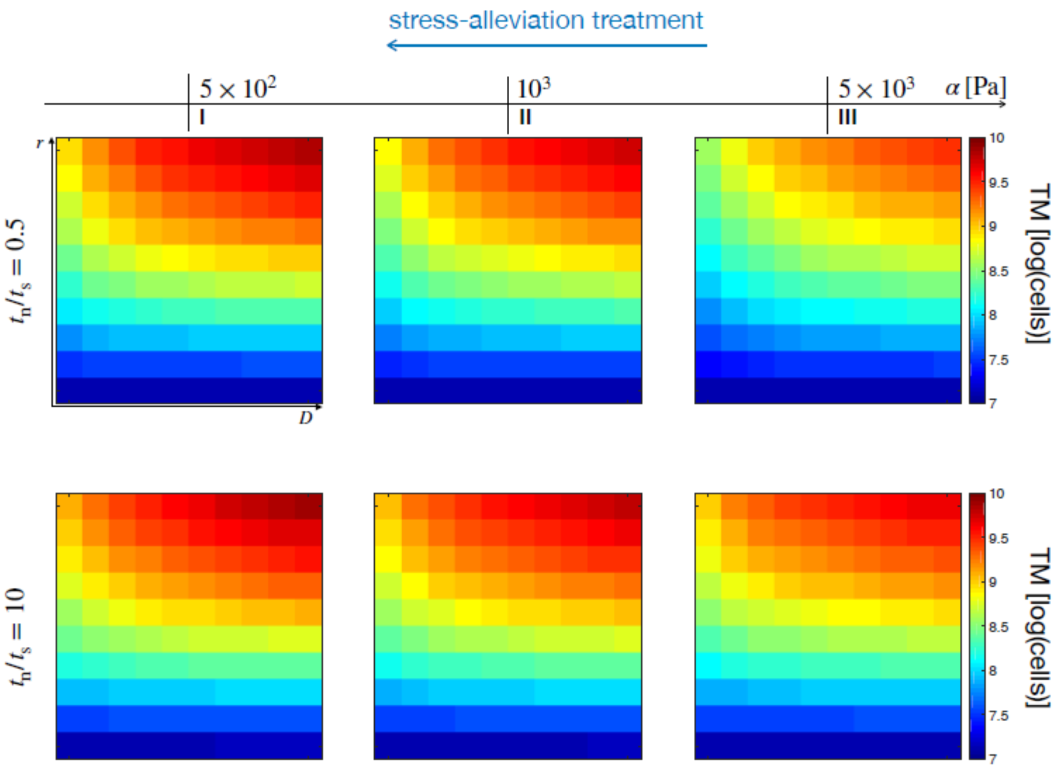

Figure S7. Simulation maps displaying the effects of chemo-mechanically induced transitions on TM. The top row shows three TM maps for different values of α at the ratio $t_n/t_s = 0.5$, whereas the bottom row displays TM values over the (D, r) space for the different stiffnesses at the $t_n/t_s = 10$ ratio. The simulations refer to the high mechanosensitivity case, i.e., $\alpha\sigma^{-1} = [10^1, 10^2]$.

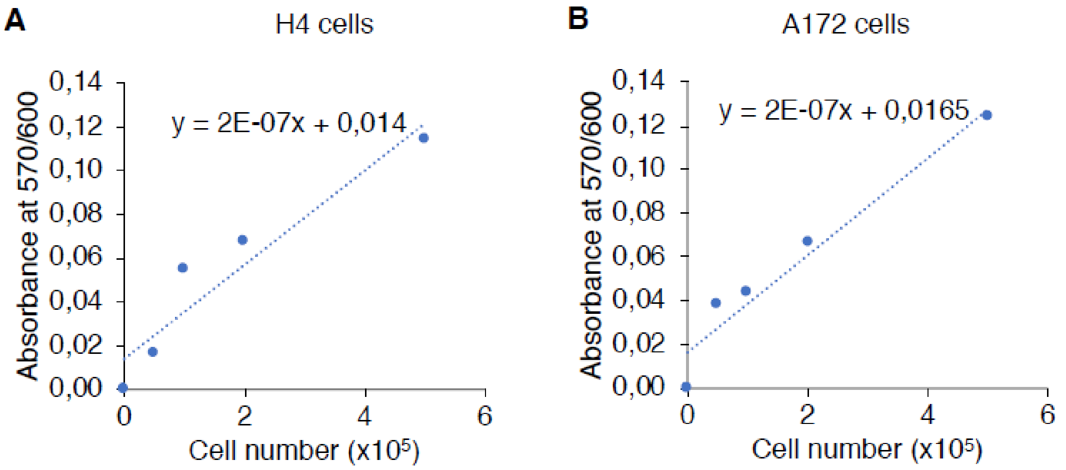

Figure S8. Calibration curves for the H4 (**A**) and A172 (**B**) cell lines for the Alamar Blue assay.

© 2019 by the authors. Licensee MDPI, Basel, Switzerland. This article is an open access article distributed under the terms and conditions of the Creative Commons Attribution (CC BY) license (http://creativecommons.org/licenses/by/4.0/).
